# NETosis in the pathogenesis of acute lung injury following cutaneous chemical burns

**DOI:** 10.1172/jci.insight.147564

**Published:** 2021-05-24

**Authors:** Ranu Surolia, Fu Jun Li, Zheng Wang, Mahendra Kashyap, Ritesh Kumar Srivastava, Amie M. Traylor, Pooja Singh, Kevin G. Dsouza, Harrison Kim, Jean-Francois Pittet, Jaroslaw W. Zmijewski, Anupam Agarwal, Mohammad Athar, Aftab Ahmad, Veena B. Antony

**Affiliations:** 1 Division of Pulmonary, Allergy and Critical Care, Department of Medicine;; 2 Department of Dermatology;; 3 Division of Nephrology, Department of Medicine;; 4 Department of Radiology; and; 5 Department of Anesthesiology and Perioperative Medicine, University of Alabama at Birmingham, Birmingham, Alabama, USA.; 6 Department of Veterans Affairs, Birmingham, Alabama, USA.

**Keywords:** Pulmonology, Molecular pathology, Neutrophils

## Abstract

Despite the high morbidity and mortality among patients with extensive cutaneous burns in the intensive care unit due to the development of acute respiratory distress syndrome, effective therapeutics remain to be determined. This is primarily because the mechanisms leading to acute lung injury (ALI) in these patients remain unknown. We test the hypothesis that cutaneous chemical burns promote lung injury due to systemic activation of neutrophils, in particular, toxicity mediated by the deployment of neutrophil extracellular traps (NETs). We also demonstrate the potential benefit of a peptidyl arginine deiminase 4 (PAD4) inhibitor to prevent NETosis and to preserve microvascular endothelial barrier function, thus reducing the severity of ALI in mice. Our data demonstrated that phenylarsine oxide (PAO) treatment of neutrophils caused increased intracellular Ca^2+^-associated PAD4 activity. A dermal chemical burn by lewisite or PAO resulted in PAD4 activation, NETosis, and ALI. NETs disrupted the barrier function of endothelial cells in human lung microvascular endothelial cell spheroids. Citrullinated histone 3 alone caused ALI in mice. Pharmacologic or genetic abrogation of PAD4 inhibited lung injury following cutaneous chemical burns. Cutaneous burns by lewisite and PAO caused ALI by PAD4-mediated NETosis. PAD4 inhibitors may have potential as countermeasures to suppress detrimental lung injury after chemical burns.

## Graphical abstract

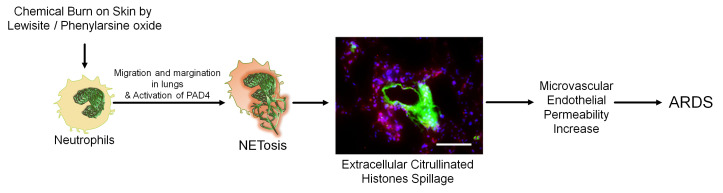

## Introduction

Acute respiratory distress syndrome (ARDS) is a heterogeneous clinical syndrome characterized by complex pathophysiological mechanisms that include dysregulated pulmonary and systemic inflammation and loss of barrier function of the alveolar-capillary membrane. There are no pathognomonic or diagnostic tests for ARDS (1) and no specific effective pharmacologic interventions available due to biological differences within the overall clinical phenotype (2). It is difficult to represent phenotype subsets due to the vast heterogeneity of ARDS (1, 3). However, ARDS endotypes can be defined by restricted molecular pathways or by differences in treatment responses or, rarely, both (4). One traditional way to subtype ARDS is by grouping patients into direct (pulmonary) versus indirect (extrapulmonary) injury. These phenotypes can be further subdivided into endotypes that can be hyperinflammatory or hypoinflammatory ARDS (3). One such cohort of extrapulmonary, hyperinflammatory ARDS is due to burn injury characterized by the presence of inflammatory biomarkers in plasma (1, 3).

In the United States, nearly half a million accidental burn injuries occur annually (5), and patients with burns involving more than 41% of total body surface area have a high probability of developing ARDS (4, 6). Importantly, patients with toxic epidermal necrolysis, a pathophysiological condition similar to skin burns, may also develop ARDS (7, 8). Burn injury–associated ARDS can be linked to hyperthermal injury or chemical toxicity of the injurious agent. There are mouse models for hyperthermal skin injury (9), but there exists a scarcity of suitable experimental models for nonthermal burns, which limits our understanding of the pathogenic mechanisms contributing to ARDS and, hence, restricts development and testing of potential therapeutic modalities for chemical burn–induced ARDS.

Lewisite, like other highly toxic arsenous chlorides, upon contact with skin inflicts nonthermal burns/blistering that, when severe, can lead to death (10). Lewisite was produced during World War II as a chemical warfare agent. Stockpiles and unknown burial sites are a potential threat for chemical terrorism and accidental exposure (11, 12). Our recent study demonstrated that cutaneous exposure to lewisite led to acute lung injury (ALI) in mice (13), but the mechanisms involved in such toxicity, including the potential contribution of activated neutrophils, need to be further elucidated. Neutrophils are the first line of defense against microbial infections (14, 15). However, neutrophil-driven proinflammatory responses have an important role in the development of lung injury in sterile inflammatory conditions such as acute blood loss associated with trauma as well as autoimmune diseases (16).

Neutrophil extracellular trap (NET) formation is a complex event that includes activation of peptidyl arginine deiminase 4 (PAD4) and concomitant citrullination of histones that leads to chromatin decondensation (17–19). NETs contribute to the severity of ARDS among patients with severe burns as evidenced by increased circulating cell-free dsDNA (20–22). In contrast, burn patients with lack of NETotic ability of neutrophils are immunosuppressed and have an increased incidence of secondary bacterial infections (23). Overall, the presence of NETs is documented in patients with extensive burns, but their direct role in the pathogenesis of ARDS or the propensity to develop ARDS has not yet been studied.

In this study, we characterize and define the underlying mechanisms of nonthermal burn–induced ALI in a nonthermal skin injury model. This study demonstrates that the development of ALI is secondary to the activation of PAD4 and the release of citrullinated histones containing NETs in the systemic circulation and lungs. Furthermore, 3D human lung microvascular endothelial cell (HLMVEC) spheroids were employed as an ex vivo functional assay to confirm the role of NETs in causing microvascular endothelial barrier impairment. Moreover, pharmacologic inhibitors of PAD4 (GSK484) were tested for their potential role in inhibiting ALI after a chemical burn injury on the skin.

## Results

### Chemical burns on the skin by lewisite trigger ALI.

**Figure 1:**
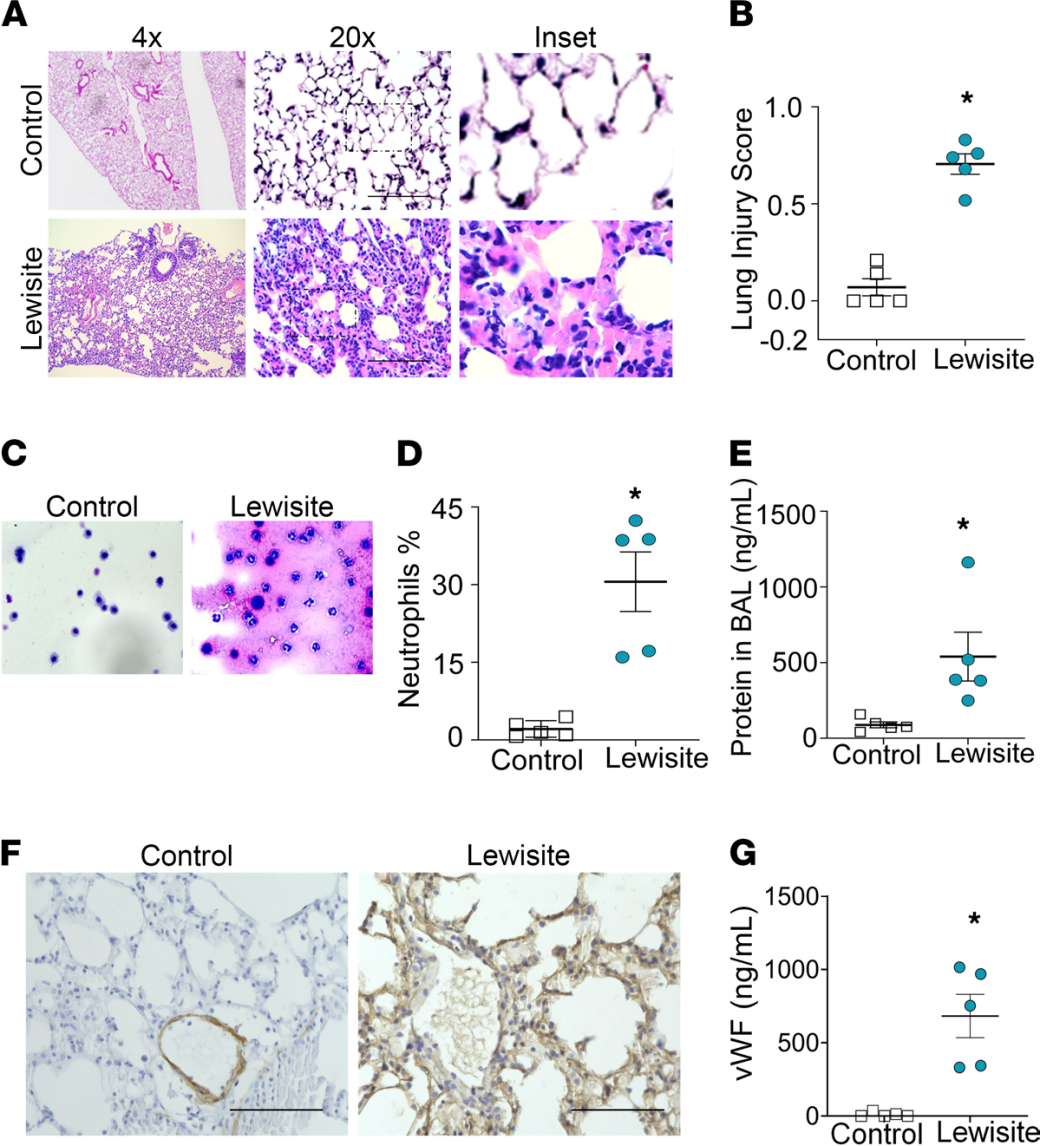
Lewisite skin burn instigates ALI. Lewisite (5.0 mg/kg) was applied to the skin of hairless Ptch1^+/−^ SKH-1 mice. Mice were sacrificed after 6 hours. (**A**) H&E images of lung sections (*n* = 5/group). (**B**) ALI scores from a blinded pathologist based on a standardized scoring system from the American Thoracic Society. Scores are continuous between 0 and 1, with 0 representing no injury and 1 representing severe ALI (*n* = 5/group). (**C**) BAL cell pellets (original magnification, ×400) stained with hematoxylin (nuclear stain). (**D**) Percentage of neutrophils counted in BAL cell pellets (*n* = 5/group). (**E**) Protein concentrations in BAL fluid (*n* = 5/group). (**F**) Immunohistochemistry staining of lung sections for vWF (*n* = 5/group). Scale bar: 100 μm. (**G**) Soluble vWF levels in serum (*n* = 5/group). All experiments were repeated 3 times. (**B**, **D**, **E**, and **G**) Each dot represents an individual mouse; error bars indicate the mean ± SEM. *P < 0.01. Statistics: 2-tailed *t* test.

Lewisite skin burns resulted in acute inflammation in the lungs at 6 hours as demonstrated by the infiltration of inflammatory cells in the parenchyma (Figure 1A) compared with controls. Lewisite-exposed mice demonstrated the presence of ALI, including features such as increased inflammatory infiltrate, thickened alveolar walls, the presence of proteinaceous material, and edematous septa (Figure 1A). The lung injury scoring system as per American Thoracic Society guidelines (24) demonstrated a high lung injury score in lewisite-exposed mice (Figure 1B). The bronchoalveolar lavage fluid (BALF) images and cell count demonstrated neutrophilia and the presence of RBCs (Figure 1, C and D). BALF from the lungs of lewisite-exposed mice had increases in the percentage of neutrophils (Figure 1D) and total protein content (Figure 1E) compared with the controls. As an extrapulmonary ARDS model, we tested if mouse lungs show any effect on vWF (25). The immunohistochemistry staining for vWF demonstrated spillage of vWF all over the lung architecture in lewisite-exposed mice (Figure 1F). The measurements of soluble vWF were increased in the serum from lewisite-exposed mice compared with the controls (Figure 1G). Taken together, single-dose exposure to lewisite on skin causes ALI in mice.

### Phenylarsine oxide skin burn induces ALI.

**Figure 2:**
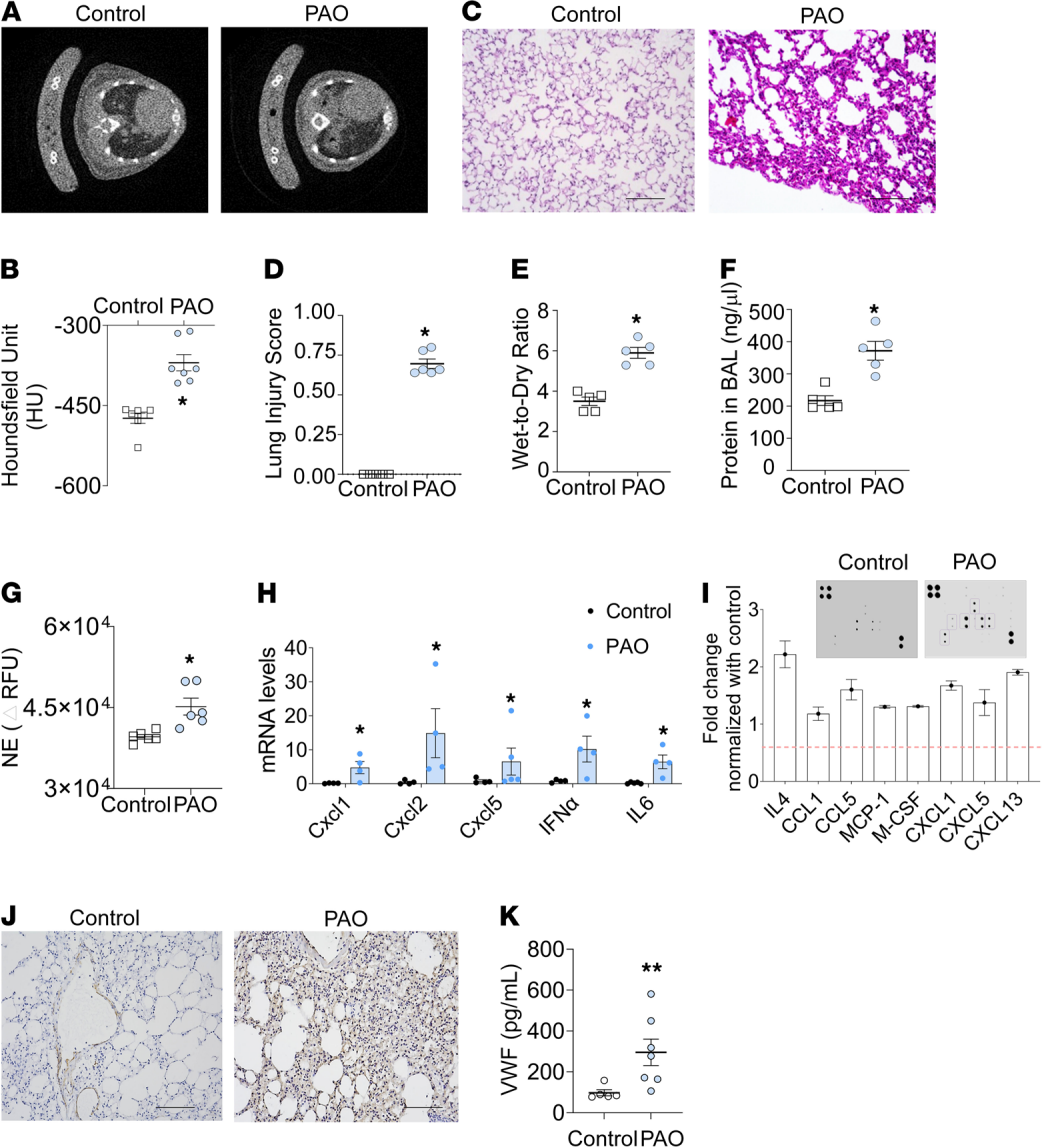
Single exposure of PAO on the skin results in ALI. PAO (150 μg/mouse in 2 cm^2^ area) was applied to the skin of shaved C57BL/6J mice. These mice were sacrificed after 6 hours. (**A**) Micro-CT scan view at thoracic vertebrae shown in control and PAO group. (**B**) Micro-CT scan analysis for density of the lung (*n* = 7/group). (**C**) H&E staining images of lung sections (*n* = 5–6/group). Scale bar: 100 μm. (**D**) ALI score (control, *n* = 5 and PAO, *n* = 6). (**E**) Wet-to-dry weight ratio of mouse lungs (*n* = 5/group). (**F**) Protein levels in BAL fluid (*n* = 5/group). (**G**) Neutrophil elastase activity in BAL fluid (*n* = 5/group). (**H**) mRNA levels for inflammatory genes in BAL cell pellets (*n* = 4/group). (**I**) Cytokine protein array for acute inflammation in whole lung lysate of control and PAO groups. The selected cytokine levels are represented in the graph after normalization with the control group (*n* = 3 for each group). (**J**) Immunohistochemistry (IHC) for vWF in lung sections (*n* = 5/group). Scale bar: 100 μm. (**K**) Soluble vWF levels in serum (*n* = 5/group). Error bars are shown as the mean ± SEM. *P < 0.05, **P < 0.01. Statistics: 2-tailed *t* test. All experiments were repeated 3 times.

Phenylarsine oxide (PAO) is an analog and degradation product of lewisite (26). Micro-CT images of lungs demonstrated increased lung density in PAO-exposed mice at 6 hours (Figure 2, A and B). The density measurements by micro-CT scans demonstrated increased lung tissue density in the PAO-exposed group (Figure 2B and Supplemental Figure 1A; supplemental material available online with this article; https://doi.org/10.1172/jci.insight.147564DS1). The lung pixel value histograms confirmed ALI (Supplemental Figure 1A). Specifically, the histograms of lung pixel values for control mouse histograms (shown in blue) peaked toward positive, whereas PAO-exposed mouse lung histograms (shown in orange) showed a negative shift (toward right) as an indicator of inflammation (Supplemental Figure 1B). PAO burns on the skin resulted in acute inflammation in the lung (Figure 2C). The lung injury score demonstrated injury in PAO-exposed mice (Figure 2D). The lung injury score correlated with an increased lung wet-to-dry ratio (Figure 2E) and increased protein content in the BALF of the PAO-exposed mice group (Figure 2F). The neutrophil elastase (NE) activity was increased in the BALF of the PAO mice group (Figure 2G). We next evaluated, as a proof of concept, a set of cytokines that are known to be involved in inflammatory response. The CXCL1, CXCL2, CXCL5, IFN-α, and IL-6 mRNA levels were upregulated in BALF cell pellets from the PAO-exposed mice group (Figure 2H). The cytokine microarray from the lung lysates of the control and PAO groups demonstrated upregulated IL-4, CCL1, CCL5, macrophage chemoattractant protein 1 (MCP-1), M-CSF, CXCL1, CXCL5, and CXCL13 protein expression exclusively in the PAO group (Figure 2I). We also observed increased expression of MCP-1, MCP-5, RANTES, TNF receptor 1, and IL-4 in the total lung lysates of PAO-exposed mice (Supplemental Figure 1C). We tested the lung sections (Figure 2J) and serum for the presence of soluble vWF (Figure 2K). The immunohistochemistry demonstrated increased soluble vWF levels in the lung sections (Figure 2J) and serum of the PAO-exposed group (Figure 2K) compared with the controls. The aforementioned data confirmed that PAO skin burns instigate ALI in mice.

### Chemical burn on skin by lewisite and PAO induces NETosis in lungs.

**Figure 3:**
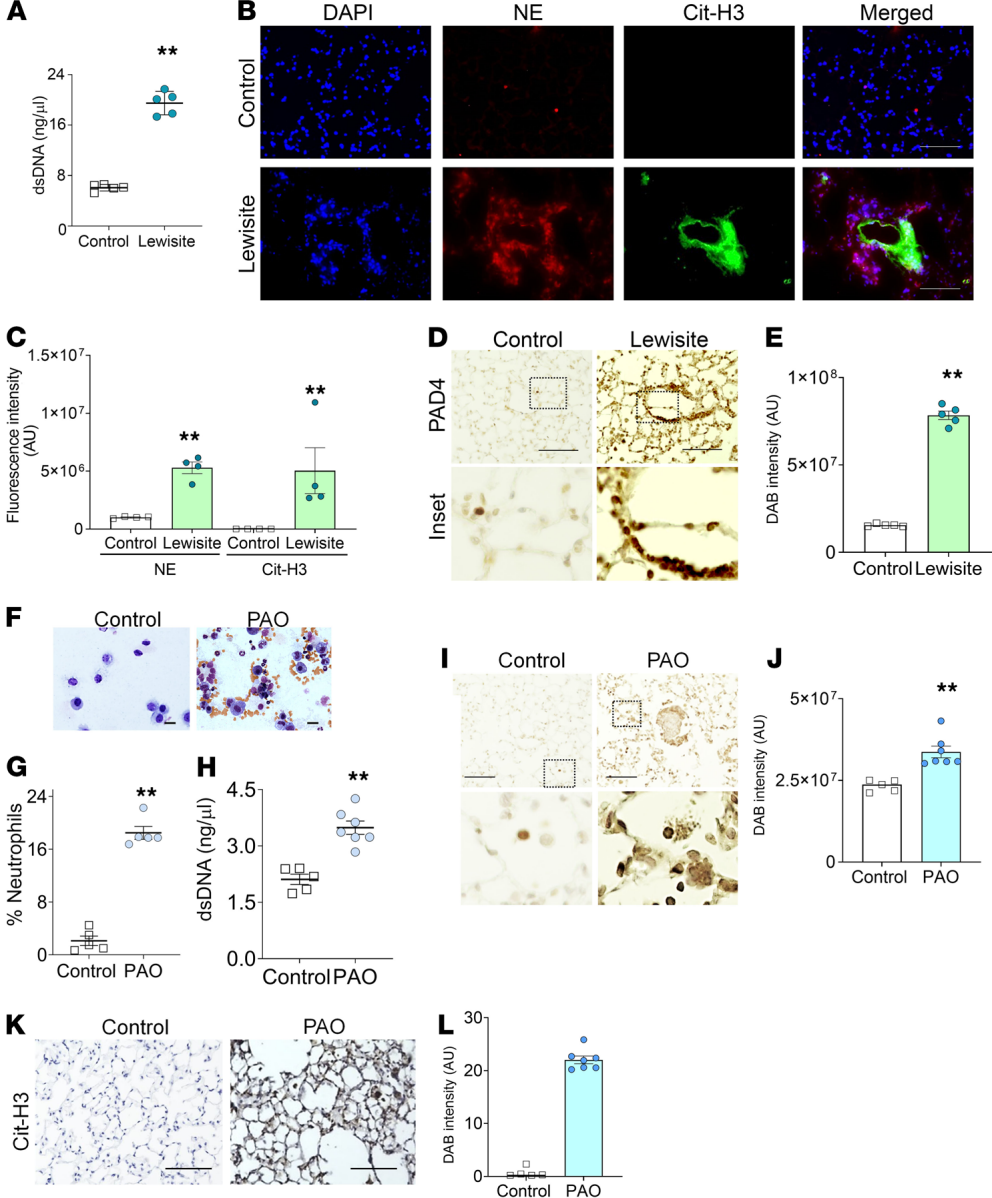
Mice exposed to lewisite and PAO on skin display NETosis in the lung. (**A**) dsDNA in the BALF of control and lewisite groups (*n* = 5/group). (**B**) Immunofluorescence staining of lung sections and (**C**) measurement of fluorescence intensity for Cit-H3 (red) and NE (green) from control (*n* = 5) and lewisite groups (*n* = 5). Scale bar: 100 μm. (**D**) IHC for PAD4 in lung sections from control and lewisite groups (*n* = 5/group). Scale bar: 100 μm. Inset: original magnification, ×200. (**E**) Measurements of DAB intensity for PAD4 from IHC images. An average measurement from 5 mice was calculated from the average of 8 images. Mice were subjected to skin exposure to PAO and sacrificed after 6 hours (**F**–**L**). (**F**) BALF cell pellets stained with hematoxylin (nuclear stain). Scale bar: 5 μm. (**G**) Percentage of neutrophils counted in BAL cell pellets (*n* = 5/group). (**H**) Measurement of dsDNA in BALF (*n* = 5/group). (**I**) IHC images for PAD4 staining (*n* = 5/group). Scale bar: 80 μm. The inset demonstrates an enlarged area of the image: original magnification, ×200. (**J**) Measurements of DAB intensity from IHC for PAD4. An average measurement from 5 mice was calculated from the average of 8 images. (**K**) IHC of the lung sections for Cit-H3. Scale bar: 100 μm. (**L**) Mean intensity of DAB for PAD4 from IHC images. An average measurement from 5 mice was calculated from the average of 8 images. All data are representations of one experiment out of 3 independent experiments. Each dot represents an individual mouse in the graphs. Data are shown as the mean ± SEM. **P < 0.01. Statistics: 2-tailed *t* test.

ALI is associated with neutrophilia (15). To detect the role of neutrophils in our extrapulmonary ALI model, we evaluated the presence of NETosis by detecting dsDNA and citrullinated histone 3 (Cit-H3) in the lungs of lewisite and PAO-exposed mice. We confirmed the presence of dsDNA in the BALF of lewisite-exposed mice (2.5-fold increase) compared with the controls (Figure 3A). Immunofluorescence staining of lung sections from the cutaneous lewisite-exposed mice demonstrated sequestration of NE-positive neutrophils (red) and spillage of Cit-H3 (green) around small blood vessels (Figure 3B). The measurements of fluorescence intensity also demonstrated increased levels of NE and Cit-H3 in the lewisite group (Figure 3C). Cutaneously exposed lewisite mice demonstrated increased levels of PAD4 in their lungs (Figure 3, D and E).

We made similar observations in PAO-exposed mice. Skin exposure to PAO increased the percentage of neutrophils (Figure 3, F and G) and cell-free dsDNA in BALF (Figure 3H). The immunohistochemistry demonstrated increased PAD4 expression levels in the lungs of PAO-exposed mice (Figure 3, I and J). The spillage of Cit-H3 over the lung architecture, specifically in the lumen and airway walls, was observed in PAO-exposed mice (Figure 3, K and L), which indicated the presence of highly NETotic interstitial neutrophils. Overall, lewisite and its analog, PAO-induced skin burns, caused NETosis in the lungs.

### PAO exposure instigates NETosis in human peripheral blood neutrophils.

**Figure 4:**
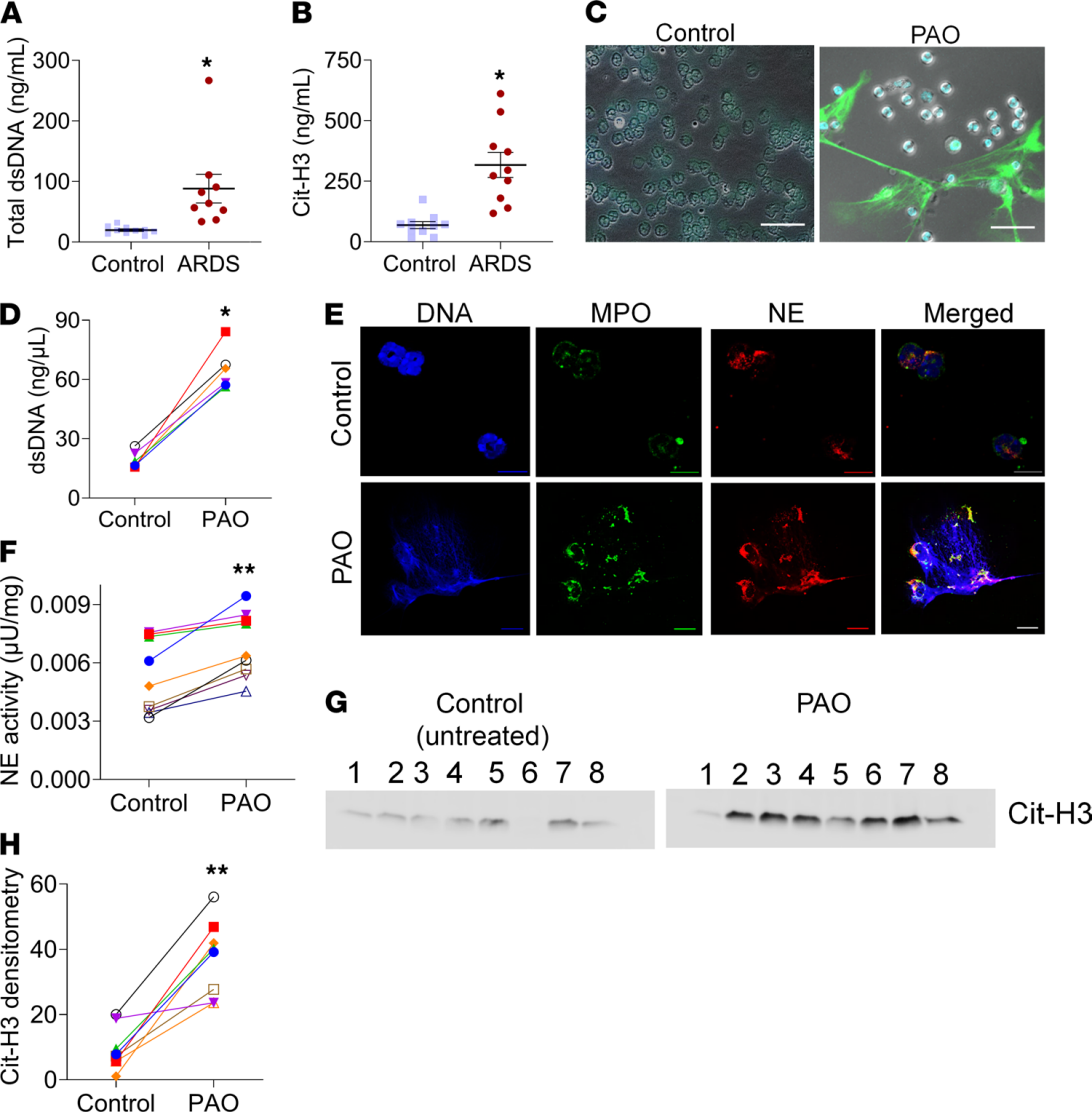
Patients with ARDS have increased NETs in plasma, and PAO treatment induces NETosis in human neutrophils. (**A**) dsDNA quantitation and (**B**) Cit-H3 in the plasma of healthy volunteers (controls) and patients with ARDS. Each symbol represents an individual subject. Data are shown as the mean ± SEM. *P < 0.05. Two-tailed *t* test. Human peripheral blood neutrophils were treated with PAO for 4 hours and analyzed for NETosis markers. (**C**) Unfixed neutrophil staining for extracellular DNA (SYTOX Green, green) and nuclear DNA (Hoechst, blue). Scale bar: 10 μm. (**D**) Measurements of dsDNA in the supernatant of neutrophils. Neutrophils were obtained from the blood of 6 healthy volunteers. (**E**) Immunofluorescence staining for myeloperoxidase (MPO) and NE in human neutrophils. Scale bar: 5 μm. (**F**) Measurements of extracellular neutrophil elastase activity. Neutrophils were obtained from the blood of 9 healthy volunteers. (**G**) Western blot analysis of NETs for Cit-H3. Each lane represents neutrophils from an individual subject. (**H**) Densitometry measurement for the Cit-H3 levels in Western blots. In graphs (**D**, **E**, and **H**), each dot represents neutrophils from an individual subject, and controls are untreated neutrophils from the same subject. *P < 0.05, **P < 0.01. Statistics: Wilcoxon’s test.

Our mouse model for chemical burn–induced ARDS demonstrated high amounts of dsDNA and NETs in their lungs. We further investigated whether patients with extensive burns have increased cell-free dsDNA in their blood. There were increased levels of dsDNA in the serum of patients with ARDS (Figure 4A), which is possible because of the abundance of cell necrosis and/or NETosis (20, 21). We investigated the presence of citrullinated histones in the plasma of patients with ARDS to evaluate the source of dsDNA and observed higher levels of Cit-H3 in their plasma compared with the controls (Figure 4B).

Due to limitations imposed by the lack of lewisite/PAO exposure human studies, we further tested the direct effects of PAO exposure on human peripheral blood neutrophils. Neutrophils exposed to PAO expelled NETs (Figure 4C) and demonstrated an abundance of extracellular dsDNA (Figure 4D). All controls used untreated neutrophils obtained from the same individual subject. As expected, the PAO-induced NETosis also demonstrated increased myeloperoxidase (MPO) levels and NE (Figure 4E), as well as extracellular NE activity in neutrophils (Figure 4F). Extracellular dsDNA (SYTOX Green) was positive for Cit-H3 (Supplemental Figure 2) from PAO-treated neutrophils. The immunoblots of NETs demonstrated increased expression of Cit-H3 in NETs from PAO-treated neutrophils compared with the controls (Figure 4, G and H). These data suggest that PAO exposure causes NETosis in human neutrophils.

### PAO induces calcium- and ER stress-dependent NETosis.

**Figure 5:**
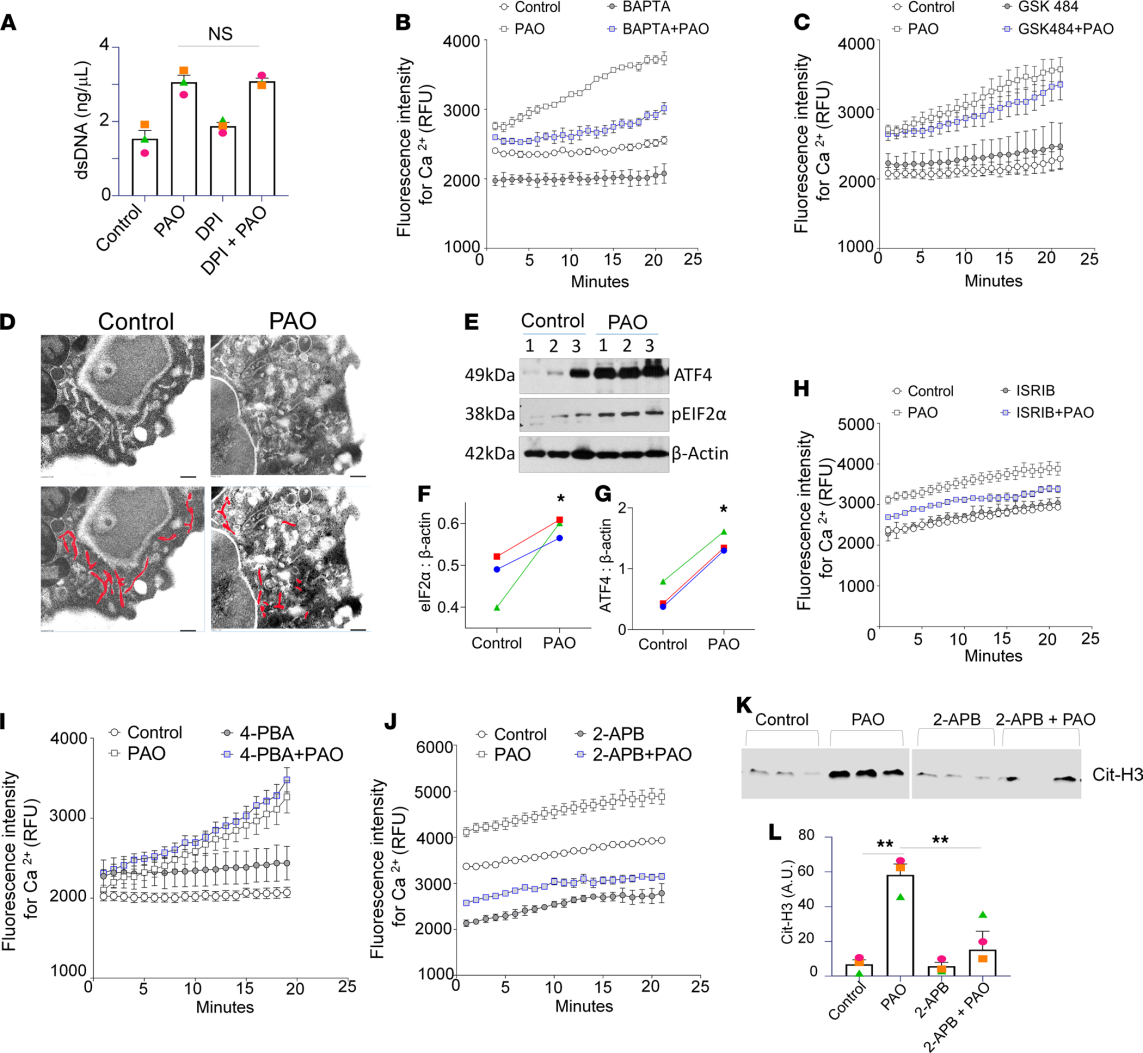
PAO induces calcium- and ER stress–dependent NETosis. (**A**) NETs’ formation is measured by dsDNA in the control, DPI-, PAO-, and DPI+PAO-cotreated neutrophil supernatants. Each dot represents neutrophils from an individual subject. Here, “*ns*” means not significant, as compared with PAO- vs. DPI+PAO-cotreated human neutrophils. Intracellular calcium flux measurements in the neutrophils isolated from human subjects loaded with calcium-sensitive dye Fluo-4 and stimulated with PAO for indicated time points in the absence or presence of (**B**) calcium chelator, BAPTA, and (**C**) PAD4 inhibitor, GSK484. All the intracellular calcium levels were measured in 3–5 individual subjects. Data are shown as the mean ± SEM. (**D**) Transmission electron microscopy images (*n* = 3). A total of 8 images were taken from each group. The endoplasmic reticulum is delineated with red lines in the lower panel. Scale bar: 2 nm. (**E**) Immunoblots of PAO-exposed neutrophils (3 hours) for ER stress marker proteins ATF4 and eIF2-α and (**F** and **G**) densitometry measurements. Individual lanes in immunoblots and symbol in graphs represent neutrophils from individual subjects. β-Actin was used as loading control. Measurements of intracellular calcium flux in Fluo-4–loaded human neutrophils stimulated with PAO in the absence or presence of (**H**) ISRIB, (**I**) 4-phenylbutyric acid (4-PBA), and (**J**) 2-APB for indicated time points. All the intracellular calcium levels were measured in 3–5 individual subjects. (**K**) Immunoblot for Cit-H3 levels in the NETs. Each lane represents lysate from an individual human subject. (**L**) Densitometry for Cit-H3 from the immunoblot. All data are shown as the mean ± SEM. *P < 0.05, **P < 0.01. Statistics: 1-way ANOVA followed by Tukey’s multiple comparisons test (**A** and **L**), 2-way ANOVA for repeated measures over period of time (**B**, **C**, and **H**–**J**), and Wilcoxon’s test (**F** and **G**).

NETosis by PAD4 activation can be NADPH oxidase 2 (NOX2) dependent and/or NOX2 independent (27). One such NOX2-independent mechanism is an intracellular Ca^2+^-dependent process (28). The NADPH oxidase inhibitor, diphenyleneiodonium chloride (DPI), showed no significant inhibitory effect on NETosis in PAO-treated neutrophils (Figure 5A). However, PAO treatment demonstrated a distinct increase in calcium influx in the neutrophils (Supplemental Figure 3). The cotreatment with the Ca^2+^ chelator, BAPTA (1,2-bis[o-amino phenoxy]ethane-N, N, N′, N′-tetraacetic acid), inhibited intracellular Ca^2+^ levels in PAO-treated neutrophils (Figure 5B). The PAD4 inhibitor GSK484 (18) demonstrated no effect on intracellular Ca^2+^ levels in PAO-treated neutrophils (Figure 5C).

Further, we investigated the source of the PAO-induced increase of intracellular calcium. The endoplasmic reticulum (ER) stores could also regulate the influx of Ca^2+^ from the extracellular space (29). To investigate PAO-mediated increased cytosolic calcium sources, we evaluated ER stress in neutrophils. The PAO-treated neutrophils displayed swelling and morphological distortion of the ER (red-colored area) (Figure 5D). The protein lysates of PAO-treated neutrophils demonstrated an increased expression of the ER stress markers activating transcription factor 4 (ATF4) and eukaryotic translation initiation factor 2A (eIF2a) (Figure 5, E–G). We utilized different types of ER stress-inhibitor cotreatments — integrated stress response inhibitor (ISRIB; Figure 5H), 4-phenylbutyric acid (4-PBA; Figure 5I), and 2-aminoethyl diphenylborinate (2-APB; Figure 5J) — to analyze the effect on intracellular calcium. The cotreatment with ISRIB and 4-PBA demonstrated no effect on intracellular Ca^2+^ upon PAO treatment (Figure 5, H and I). However, cotreatment with 2-APB demonstrated decreased levels of intracellular Ca^2+^ (Figure 5J). The immunoblot analysis also demonstrated decreased levels of Cit-H3 in the isolated NETs (Figure 5, K and L). Overall, the ER stores were associated with an upsurge of intracellular Ca^2+^ levels and NETosis.

### PAO-induced NETs disrupt the barrier function of HLMVEC monolayers and spheroids.

**Figure 6:**
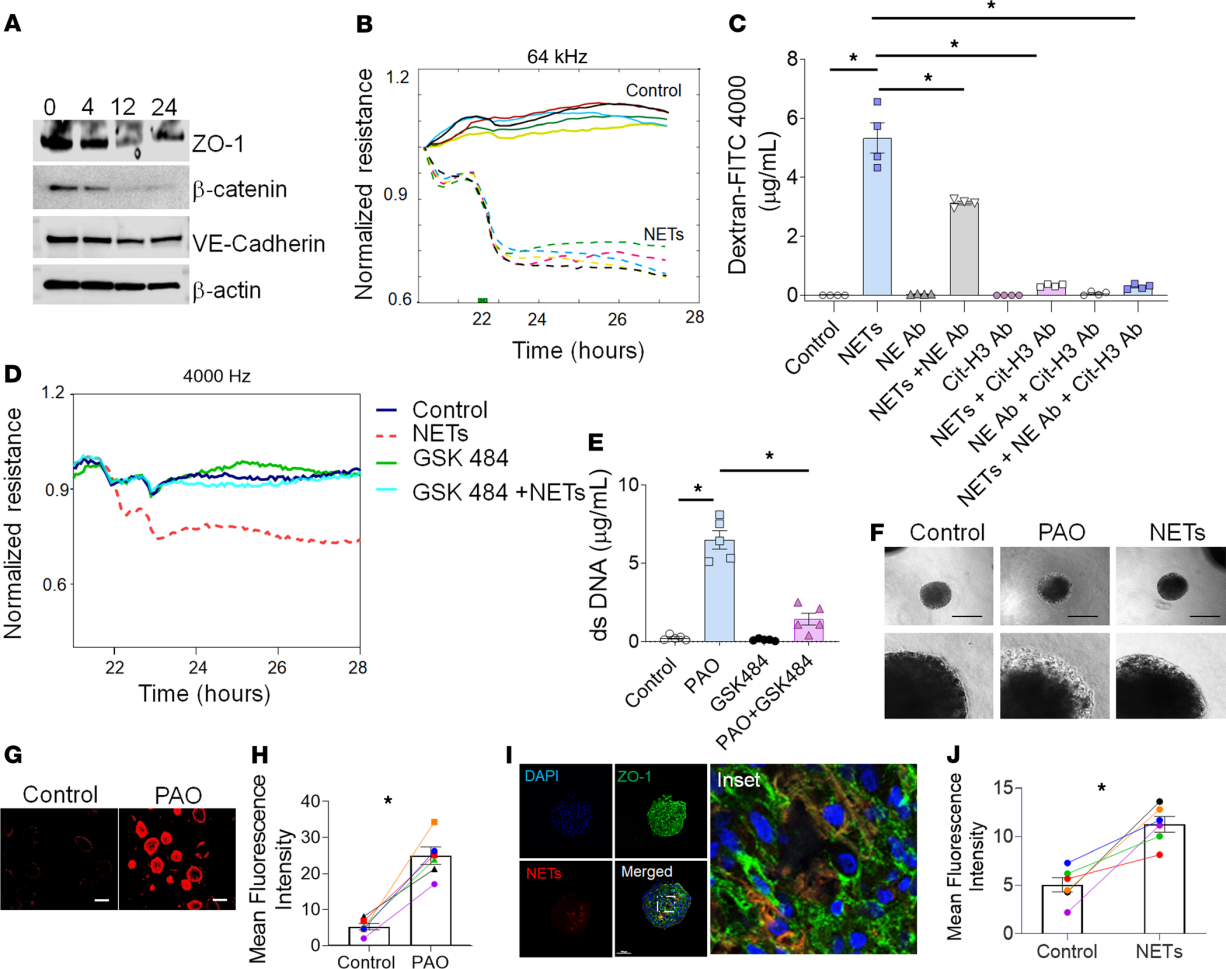
PAO-induced NETs disrupt barrier function in HLMVEC monolayers and spheroids. (**A**) Representative immunoblot of ZO-1, VE-cadherin, and β-catenin after treatment with NETs isolated from human neutrophils after PAO treatment for indicated time points (*n* = 5). (**B**) Comparisons of normalized resistance of the HLMVEC monolayer upon stimulation with NETs. Each line represents NETs from an individual human subject. The stimulation time point is set as *t* = 0. (**C**) FITC-Dextran 4000 permeability assay. HLMVEC monolayers treated with vehicle (control), NETs, NE antibody, NE antibody and NETs, Cit-H3 antibody, Cit-H3 antibody and NETs, and Cit-H3 antibody, NE antibody, and NETs for 24 hours. (**D**) Representative data for comparisons of normalized resistance upon stimulation with NETs derived from controls (untreated), PAO, GSK484, and GSK484- and PAO-cotreated neutrophils in HLMVEC monolayers (*n* = 3 subjects). (**E**) Measurements of extracellular dsDNA levels in the supernatant of the control, PAO, GSK484, and GSK484- and PAO-cotreated neutrophils (*n* = 4). (**F**) Phase contrast images of spheroids treated with PAO or NETs (*n* = 3). Original magnification (lower panel), ×75. Images (**G**) and graph (**H**) for dextran permeability assay in primary HLMVEC spheroids. Scale bar: 200 μm. (**I**) Fluorescence images showing the expression of tight junction markers, ZO-1 (green), and extracellular dsDNA (NETs) (TOTO 3, red). Nuclei of spheroids were stained with Hoechst dye (blue). Scale bar: 100 μm. Inset showing magnified fluorescence image showing ZO-1 expression. Original magnification, ×15,000. (**J**) Dextran permeability assay in primary HLMVECs. All data are shown as the mean ± SEM. *P < 0.05. Statistics: 1-way ANOVA followed by Tukey’s multiple comparisons test (**C** and **E**) and Wilcoxon’s test (**H** and **J**).

Barrier disruption often stems from compromised interendothelial tight junctions, resulting in the formation of gaps between normally adjoining cells (30). Treatment of HLMVECs with NETs isolated from PAO-exposed neutrophils decreased the levels of zonula occludens 1 (ZO-1), vascular endothelial-Cadherin (VE-Cadherin), and β-catenin in HLMVEC monolayers (Figure 6A and Supplemental Figure 4). NET treatment on HLMVEC monolayers decreased the barrier function at 1 to 2 hours as compared with the controls (Figure 6B). Cotreatment with anti-NE or anti–Cit-H3 reinstated barrier function when compared with only NET-treated and control HLMVEC monolayers. The improved barrier function of HLMVEC monolayers was more efficient in NETs neutralized with anti–Cit-H3 as compared with anti–NE-neutralized NETs (Figure 6C).

NETs from PAO-treated neutrophils decreased barrier function, whereas NETs from GSK484- and PAO-cotreated neutrophils demonstrated no change in barrier function as compared with only GSK484-treated and control neutrophil NETs (Figure 6D). As expected, GSK484 decreased NETosis in PAO-exposed neutrophils compared with those that only received the PAO treatment (Figure 6E).

PAO- and NET-treated HLMVEC spheroids demonstrated a differential refractive index of more permeabilized layers visible as different superficial radiance/corona around the spheroids. PAO treatment causes thicker radiance on the circumference of the spheroids as compared with the spheroids treated with NETs, demonstrating increased permeability. The controls did not show any peripheral radiance (Figure 6F). PAO treatment increased uptake of tetramethylrhodamine-dextran (TRITC-dextran) in spheroids (Figure 6G) compared with the controls. PAO-treated spheroids demonstrated increased mean intensity for TRITC-dextran compared with the controls (Figure 6H). The immunofluorescence staining (TOTO 3, a carbocyanine dimer stain, red) of NET-treated spheroids demonstrated increased paracellular gaps, decreased ZO-1 expression (green) (Figure 6I), and increased mean fluorescence intensity for TRITC-dextran after 24 hours (Figure 6J). Thus, NETs, and specifically Cit-H3, disrupted the barrier function of HLMVECs.

### PAD4 is essential for NETosis-induced ALI in PAO cutaneous burn murine model.

**Figure 7:**
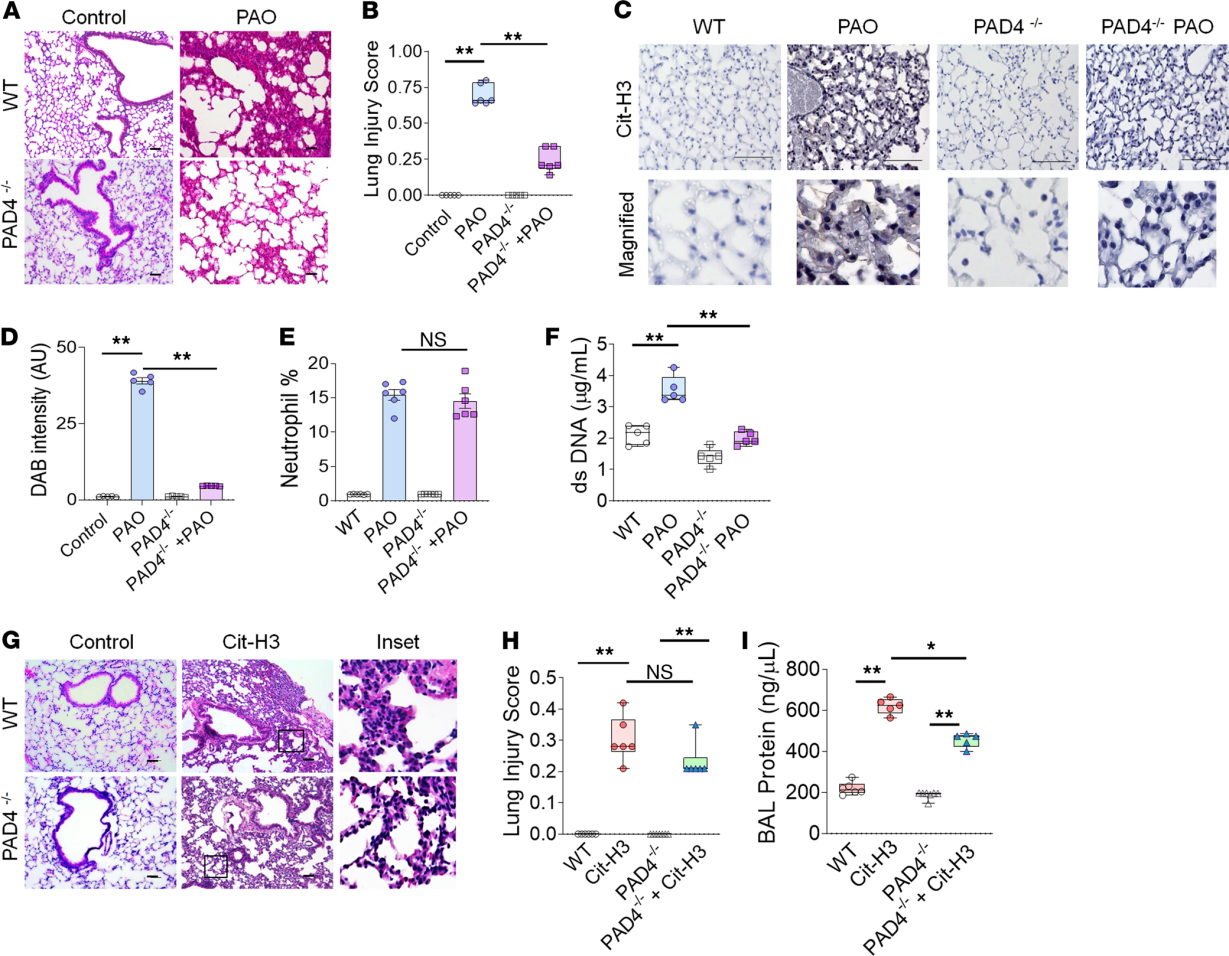
PAD4^–/–^ mice have diminished ALI following cutaneous exposure to PAO. WT and PAD4^–/–^ mice were exposed to PAO on skin and sacrificed after 6 hours. (**A**) H&E staining of lung sections (*n* = 5/group). Scale bar: 100 μm. (**B**) Lung injury score (*n* = 5/group). (**C**) IHC staining of lung sections for Cit-H3 (*n* = 5/group). Scale bar: 100 μm. Lower panel original magnification, ×200. (**D**) Measurements of DAB intensity from IHC for Cit-H3. An average measurement from 5 mice was calculated from the average of 8 images. (**E**) Percentage of neutrophils in BAL fluid (*n* = 5–6 /group). (**F**) Measurement of dsDNA levels in BAL fluid. WT and PAD4^–/–^ mice were treated with Cit-H3 peptides (25 μg/mouse, intratracheally) and sacrificed after 12 hours. (**G**) H&E staining of lung sections (*n* = 5/group). Scale bar: 100 μm; inset original magnification, ×100. (**H**) Lung injury score (*n* = 5/group) and (**I**) protein in BAL fluid (*n* = 5). Statistics: (**B**, **F**, **H**, and **I**) box-and-whiskers plots display median, and whiskers show maximum to minimum value. Each symbol represents an individual mouse. *P < 0.05, **P < 0.01. Data were analyzed by using 1-way ANOVA followed by Tukey’s multiple comparisons test. In graphs (**D** and **E**) data are shown as the mean ± SEM. **P < 0.01.

PAD4 is essential for chromatin decondensation by citrullination of histones in NETosis (18). To investigate the pathological significance of enhanced PAD4 activation, B6.Cg-Padi4^tm1.1Kmow^/J (PAD4^–/–^) mice were exposed to PAO on the skin. PAD4^–/–^ mice had an attenuated lung injury compared with PAD4^+/+^ (WT) mice (Figure 7, A and B). The lungs of PAO-exposed PAD4^–/–^ mice had reduced Cit-H3 spillage (Figure 7, C and D). To determine whether the attenuation of PAO-inflicted ALI in PAD4^–/–^ mice was related to reduced neutrophil influx or lack of NETosis, we analyzed BALF cells. There was no significant difference in neutrophil counts in the BALF of PAO-exposed PAD4^–/–^ and WT mice (Figure 7E), but PAO-exposed PAD4^–/–^ mice exhibited lower levels of dsDNA compared with PAO-exposed WT mice (Figure 7F). WT and PAD4^–/–^ controls had no significant difference in the percentage of neutrophils and dsDNA levels (Figure 7, E and F). These data confirmed that the lack of PAD4 has no effect on neutrophil influx but inhibits NETosis that results in attenuated PAO-induced ALI. Further, we investigated the individual role of Cit-H3 in lung injury. Cit-H3 is an essential component of NETosis, and our data demonstrated that the intratracheal administration of Cit-H3 resulted in lung injury in WT and PAD4^–/–^ mice (Figure 7, G and H). As expected, the protein leak correlated positively with the degree of lung injury (Figure 7I). Overall, these data suggested that PAD4 is essential for the NETosis-mediated injury in the cutaneous exposure model of PAO-induced ALI, and the PAD4 activity product Cit-H3 acts as a spearhead to cause lung injury.

### Pharmacological inhibitor of PAD4 attenuates PAO skin burn–induced ALI.

**Figure 8:**
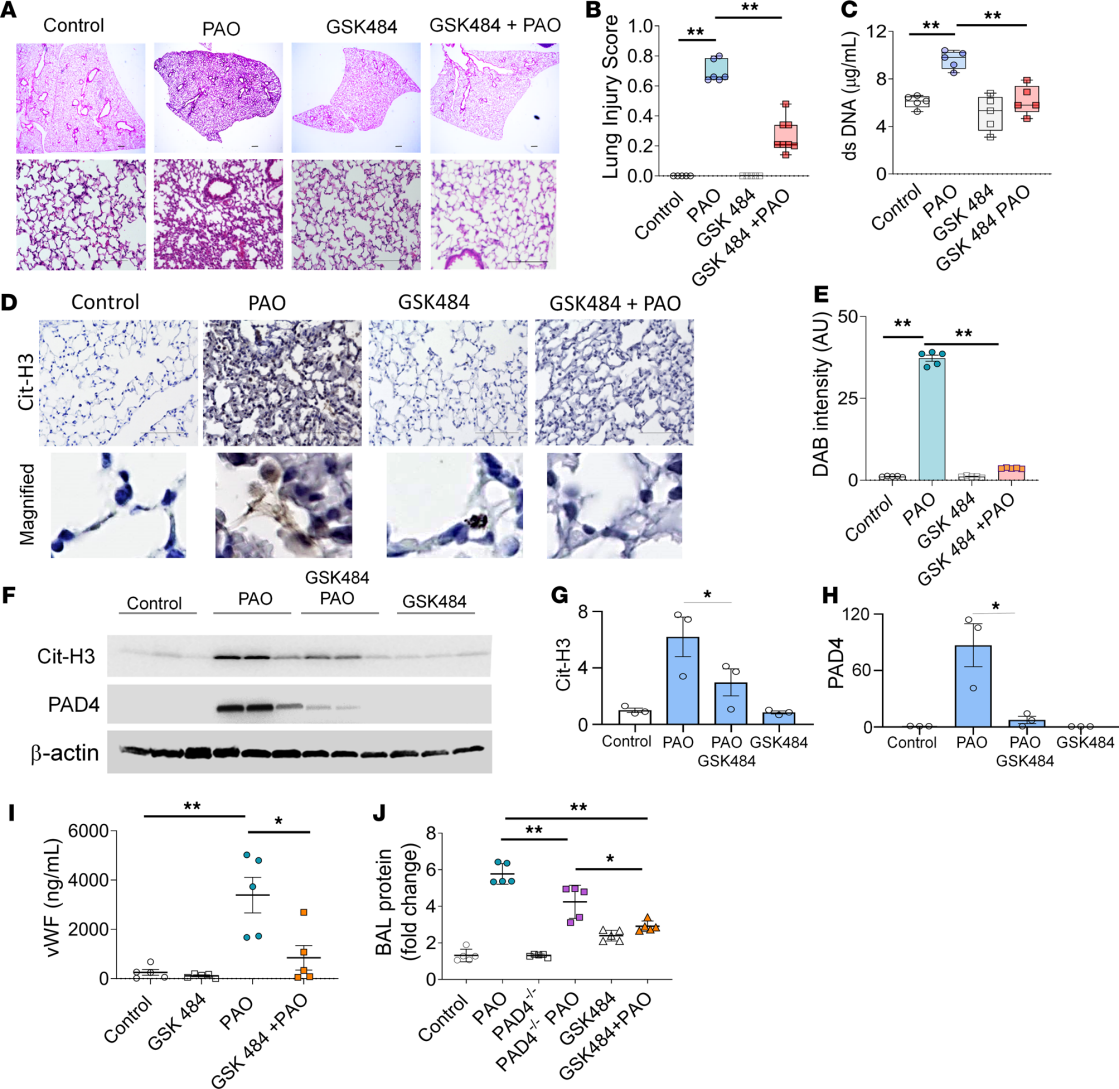
GSK484-attenuated PAO-mediated ALI in mice. WT mice were injected i.p. with GSK484 before the exposure to PAO on skin and sacrificed after 6 hours. (**A**) H&E staining, (**B**) lung injury score (*n* = 5/group), and (**C**) dsDNA in BAL fluid control, GSK484, PAO, and PAO and GSK484 groups (*n* = 5/group). Scale bar: 100 μm. (**D**) IHC staining of lung sections for Cit-H3 from control, GSK484, PAO, and PAO and GSK484 groups. Scale bar: 100 μm. Lower panel original magnification, ×2500. (**E**) Measurements of DAB intensity from IHC for Cit-H3. An average measurement from 5 mice was calculated from the average of 8 images. (**F**) Soluble vWF levels in serum (*n* = 5/group). (**G**) Western blot analysis for PAD4 and Cit-H3 expression in lung homogenates (*n* = 3). (**H**) Western blot densitometry for Cit-H3 and PAD4. The Cit-H3 and PAD4 densitometry was normalized with β-actin. (**I**) vWF levels in the serum (*n* = 5/group). (**J**) Fold changes in the protein leak in BAL fluid from control, PAO, PAD4^–/–^, PAD4^–/–^ PAO, GSK484, and GSK484 and PAO groups (*n* = 5–6 mice/group). Statistics: (**B** and **C**) box-and-whiskers plots display median, and whiskers show maximum to minimum value. Each symbol represents individual mouse. **P < 0.01. Data were analyzed by using 1-way ANOVA followed by Tukey’s multiple comparisons test. In graphs (**E** and **G**–**J**), data are shown as the mean ± SEM. *P < 0.05,**P < 0.01.

To further validate the importance of PAD4 activation and to test the potential role of PAD4 inhibitors as potential therapies, we employed pretreatment with GSK484 in our experimental murine model of nonthermal burn-related extrapulmonary ARDS. GSK484 pretreatment reduced ALI in PAO-exposed mice (Figure 8, A and B). GSK484 pretreatment also reduced cell-free dsDNA in BAL of PAO-exposed mice (Figure 8C). However, the number of neutrophils was unaffected in BAL fluids from GSK484-pretreated PAO-injured mice. Because of the suppression of PAD4 activity, Cit-H3 levels diminished in GSK484-pretreated, PAO-injured mice compared with the PAO-exposed mice (Figure 8, D and E). Lung homogenates from GSK484-pretreated, PAO-exposed mice demonstrated decreased PAD4 expression and reduced Cit-H3 (Figure 8F). Densitometry of immunoblots demonstrated that the PAO-treated mice lung lysates consisted of the highest expression of PAD4 and Cit-H3 compared with the control and GSK484- and PAO-treated mouse lungs (Figure 8, G and H). The endothelial injury marker, soluble vWF, was also diminished in GSK484-treated, PAO-exposed mice (Figure 8I).

Finally, we measured microvascular permeability changes in the mouse lungs in the aforementioned models of ablated PAD4 activity in terms of the fold change of the protein leak in BALF. The BALF from the PAO-exposed WT group exhibited the highest protein leak compared with the PAD4^–/–^ controls and only GSK484-treated controls (Figure 8J). PAD4^–/–^ mice exposed to PAO demonstrated a decreased protein leak compared with PAO-exposed WT mice. Similarly, inhibition of PAD4 activity by GSK484-pretreated, PAO-exposed mice had decreased total protein levels compared with PAO-exposed WT mice. GSK484 pretreatment in the PAO-exposed mice suppressed protein leaks more efficiently than in the PAO-exposed PAD4^–/–^ mice group. Overall, our results demonstrate that inhibition of PAD4 activity by GSK484 attenuates PAO skin burn–induced ALI.

## Discussion

ARDS in patients with extensive burns is one of the most common causes of mortality (2, 4, 6, 31). The local and systemic inflammatory response to dermal burn injury is extremely complex and results in both local burn-induced tissue damage and deleterious systemic effects on other organ systems distant from the burn area itself. In chemical burn injury, dermal destruction through chemical reactions occurs rather than hyperthermic injury (32). Nonthermal chemical burns can cause ARDS (7). The molecular pathways for chemical burns related to ALI are not clear. Here, our potentially novel murine model for skin nonthermal chemical burn–induced ALI indicates a critical and essential role of PAD4. Moreover, our studies confirm microvascular endothelial injury directly by PAO and by NETs, a product of PAD4 activity, in 3D spheroids developed from primary human lung microvascular endothelial cells.

Subjects with extensive dermal burns have increased cell-free DNA (20, 21), which is a prognostic marker for mortality (20, 33). Our studies of human neutrophils demonstrate that PAO treatment increased PAD4 activity and results in the release of cell-free Cit-H3–decorated dsDNA, which are distinct characteristics of NETosis (34, 35). NETosis is the eventual result of PAD4 activity by NADPH oxidase–dependent or –independent pathways, depending on the activity-initiating stimulus (27, 36, 37). Rapid NETosis is usually associated with increased Ca^2+^ influx in the cell (27, 37). Ca^2+^ promotes the bioactive conformation of PAD4 and increases its activity 10,000-fold (28) for citrullination of histones and consequent expelling of NETs (17, 18). PAO treatment of human neutrophils demonstrated a rapid cytosolic increase of calcium influx, suggesting NETosis activation by Ca^2+^ influx rather than NADPH oxidase activation. These results are in agreement with other studies showing that PAO inhibits NADPH oxidase activity to bind with reactive oxygen species (38, 39). Furthermore, chelation of Ca^2+^ by BAPTA treatment inhibited PAO-induced NETosis, which also added to the evidence of intracellular Ca^2+^-biased NETosis pathways (27). In contrast, a study showed no changes in cytosolic Ca^2+^ levels in absence of exogenous Ca^2+^ in rat neutrophils upon treatment with PAO (40). This discrepancy in results may be due to the hormesis effect of PAO toxicity with different doses in neutrophils.

The lumen of the ER is an intracellular source of cytosolic calcium (41). Previously, our group has demonstrated that unfolded protein response signaling regulates PAO toxicity in the skin and kidney (42, 43). Our data from human blood neutrophils and mouse lungs demonstrated induction of ER stress by PAO exposure. Activated PKC generates inositol trisphosphate (IP3), which diffuses rapidly within the cytosol, binds ER-located IP3 receptors, and allows the deletion of the Ca^2+^ storage in the ER lumen (44). The emptying of Ca^2+^ from the ER triggers external Ca^2+^ entering into the cell through the slow activation of store-operated Ca^2+^ channels (44). Treatment with ER stress inhibitors, such as 2-APB, decreased dsDNA release, but 4-PBA and ISRIB (inositol-requiring transmembrane kinase/endoribonuclease 1α and PRKR-like ER kinase inhibitors) showed no effect on extracellular dsDNA expulsion. The inhibition of IP3 receptors by 2-APB (45) inhibited PAD4 and NETosis. These studies demonstrated the specific role of ER-luminal calcium stores in the rapid generation of NETs by PAO through activating PAD4.

NETs disrupt the endothelial cell barrier function in sterile inflammatory injuries and cause organ damage (35, 46). NETs can contribute to permeability increases by expelling NE and/or citrullinated histones in the vicinity of the endothelium (30). Nevertheless, the PAO-induced NETs were similar to ionomycin (inducer of Ca^2+^ influx-mediated NETs) in terms of disrupting the barrier function of HLMVECs (Supplemental Figure 5). The detailed experimental approach demonstrated that the neutralization of NE in PAO-induced NETs partially suppressed the permeability changes in HMVEC monolayers, whereas antibody neutralization for extracellular Cit-H3 diminished the permeability changes in the HLMVEC monolayer. Histones have an affinity to bind with vWF on endothelial cells (47) and NETs (48, 49). There is a direct correlation of increased NETs and vWF levels in the plasma of subjects with burns (21). Furthermore, elevated serum vWF is correlated with the development of ARDS in patients with extensive burns (50). The neutralization of Cit-H3 disrupts this binding, thus inhibiting the effect of Cit-H3 on the endothelial barrier. HLMVEC spheroids were used as an aid to overcome the lack of accessibility or well-recorded human case studies of lewisite exposure. When compared with primary HLMVEC monolayers, we observed that the monolayers have a significant decrease in barrier function within 2 hours after NET treatment (*P* < 0.05), whereas the spheroid permeability changes were evident after 24 hours of incubation. The direct toxicity of PAO due to its affinity to the thiol group resulted in faster and more pronounced permeability changes in HLMVEC monolayers and spheroids (51). These differences are due to the controlled diffusional effect to mass transport of PAO or NETs over the surface of endothelial cells similar to in vivo conditions. Spheroids recapitulate the curvature effect of blood vessels that provides circumferential deposition of extracellular matrix and corresponding cytoskeletal protein arrangements. This mimics the arrangement of tight junctions of the microvascular endothelial cells in the lungs and contributes to the delayed permeability changes in spheroids compared with monolayers. The use of primary HLMVEC spheroids provides a viable human model for studying sex-biased or individual-to-individual variations in the effect of formidable toxic substances.

The increase in inflammation and NETosis are emblematic features of ALI (14). Our in vivo model of a chemical dermal burn by lewisite and PAO elicits an increase in proinflammatory cytokines in lungs and NETosis as evidence of ALI. In normal conditions, the transmigration and margination of neutrophils require shape deformation and slowing down of their movements in order to pass through microvascular capillaries due to their smaller diameter compared with their own size (52). Our in vivo model demonstrated neutrophilic sequestration and presence of NETs in the lungs, which is similar to ARDS after PAO or lewisite exposure; the decreased transit time of activated and NETotic neutrophils in pulmonary circulation led to sequestration and disruption of the pulmonary microvasculature. The increased protein leak and NETosis within 6 hours of skin exposure demonstrated rapid alterations in pulmonary microvasculature in the lungs. The presence of elevated vWF levels in the plasma and lung tissue also confirmed endothelial injury (3, 25). The leakage of vWF is also supported by our recently published observation of decreased pulmonary gas exchange in the lewisite-exposed mice. Taken together, the proinflammatory cytokine profile, presence of NETs in lungs, and increased soluble vWF in the plasma demonstrated the hallmark characteristics of indirect ARDs with a hyperinflammatory endotype (3).

Furthermore, lewisite- and its analog PAO exposure–associated ALI can be due to the direct toxicity of the chemical itself, albeit the superimposition of NETosis by activated neutrophils markedly enhances the effect on the permeability of the pulmonary microvasculature. This suggests that the depletion of neutrophils may suppress the injury, although there is considerable debate about any positive effect of neutrophil depletion that may be negated by its divergent role in tissue injury and repair (14, 53–55). As an alternative, we specifically targeted NETosis by using genetic and pharmacological approaches. The complete abrogation of PAD4 minimized the effects of NETosis and citrullination of histones upon PAO exposure and demonstrated attenuated ALI in PAD4^–/–^ mice. The pharmacological inhibition of PAD4, by using GSK484 treatment before skin exposure to PAO, also diminished overall lung injury. Furthermore, the suppressed lung injury correlated positively with decreased vWF levels in the plasma (50) of GSK484-treated mice exposed to PAO. These strategies also worked in the other ALI models, including sepsis and the cecal ligation and puncture–induced ALI model (56–58). Although our study indicates that both genetic and pharmacological inhibition of PAD4 protects from PAO-induced lung injury, GSK484 treatment demonstrated improved efficacy at inhibiting PAO-induced acute lung injury as compared with PAD4^–/–^ mice. PAD4 is not only required for NETosis in neutrophils but also crucial in differentiation, development, and apoptosis through gene regulation (59–61), whereas GSK484 treatment inhibited/suppressed the immediate release of NETs after PAO exposure. The amount of NETs has a negative impact on the barrier function of the HLMVECs (Supplemental Figure 6). PAD4-KO mice lack not only the formation of NETs but also the global citrullination of other proteins that could be important for homeostasis in the lung (62). Based on the aforementioned observations, we reason that the specific pharmacologic inhibition of PAD4 activity during inflammation prevented ALI more effectively than overall PAD4 abrogation.

Our in vitro data on the direct effect on Cit-H3 in barrier function disruption in HLMVEC monolayers directed us to assess the role of Cit-H3, a product of PAD4 activity, on permeability changes in the microvasculature of mouse lungs. As expected, Cit-H3 alone was enough to cause ALI. Our data are in accordance with other studies showing extracellular histones can themselves trigger sterile inflammation (35, 46). Moreover, PAD4-mediated citrullination of these histones promotes dysfunction of the endothelial barrier (63).

In summary, nonthermal cutaneous burn injuries can prompt NETosis in the lungs to cause ALI by amplified PAD4 activity in peripheral neutrophils. The extracellular Cit-H3 from NETs enhances the permeability of the microvascular capillary system in the lung, specifically by targeting vWF. Characterization of our mouse models of lewisite/PAO skin burn injury also suggests that they are a potential model for further study of extrapulmonary ARDS phenotype with a hyperinflammatory endotype. Furthermore, our study suggests that the PAD4 inhibitor GSK484 can potentially function as a countermeasure to inhibit the initiation of ALI.

## Methods

### Skin burn model of lewisite and PAO.

Lewisite exposures on Ptch1^+/−^ SKH-1 mice were performed at MRIGlobal (Kansas City, Missouri, USA) since access to or synthesis of these dangerous chemicals in the United States is only allowed by agencies that have adequate facilities for their safe storage, handling, use, and decontamination. Ptch1^+/−^ SKH-1 hairless mice were generated by backcrossing Ptch1^+/−^ C57BL/6J (The Jackson Laboratory) into SKH-1 (Charles River Laboratories) background. PAO (MilliporeSigma, catalog P3057) exposures were performed on PAD^–/–^ (B6.Cg-Padi4^tm1.1Kmow^/J) and PAD4^+/+^ mice (C57BL/6) at the University of Alabama animal facility. PAD^–/–^ and PAD4^+/+^ mice were purchased from The Jackson Laboratory. Lewisite or PAO was applied once topically on the dorsal skin of mice on a 2.56 cm^2^ skin area, as described (13). Briefly, mice were anesthetized with 100 mg/kg of ketamine and 5–7 mg/kg of xylazine injection i.p. and 0.05–0.1 mg/kg buprenorphine as an analgesic. Lewisite was dissolved in 30 μL of ethanol and applied at 5 mg/kg doses. Shaved 8- to 12-week-old, male and female C57BL/6 mice were treated with PAO topically (150 μg/mouse diluted in 30 μL ethanol and applied over 2.56 cm^2^ skin area). After 6 hours of exposure, buprenorphine (0.05–0.1 mg/kg) was administered i.p., followed by ketamine (150 mg/kg) before further processing (*n* = 5/group). GSK484 (10 mg/kg body weight, Cayman Chemicals) was administered i.p. 30 minutes before the PAO exposure on the skin. Mice were sacrificed after 6 hours of PAO exposure. All animal protocols were approved by the Institutional Animal Care and Use Committee at the University of Alabama at Birmingham (UAB).

### In vivo administration of Cit-H3.

Cit-H3 protein (25 μg/mouse) (Cayman Chemical) was delivered intratracheally by the oropharyngeal route to WT and PAD4^–/–^ mice (8–12 weeks old, male and female). The animals were sacrificed 6 hours after treatment.

### Micro-CT imaging of mouse lungs.

Micro-CT imaging on PAO-exposed mice was done as described (64). Briefly, micro-CT imaging was conducted using X-SPECT (Gamma Medica, Inc), a small-animal SPECT, and a CT scanner. The spatial resolution of the CT images was 150 μm, while the field of view was 79 mm. The entire lung region was semiautomatically segmented using a lab-made software based on LabVIEW v17.0 (NI), and the histogram features of lung tissue density in Hounsfield Units including mean, SD, and skewness were retrieved together with the entire lung volume. The histogram feature extraction was also implemented using a lab-made software based on LabVIEW v17.0.

### ELISA for NE, Cit-H3, and soluble vWF measurements.

Lungs, BALF, BALF cells, and plasma were obtained from animals of each group (13) and assessed for NE activity (Abcam, catalog ab204730), dsDNA (Quant-iT PicoGreen assay, Invitrogen, Thermo Fisher Scientific, catalog P7589), Cit-H3 (Cayman Chemical, catalog 501620), and vWF (Abcam, catalog ab229397) measurements in BALF and/or plasma or tissue (as mentioned in Results) according to the manufacturers’ specifications.

### Cytokine antibody arrays.

Whole blood was collected from vehicle- and PAO-exposed mice in BD Microtainer tubes with a serum separator additive and serum was obtained by centrifugation. Serum samples (100 μL) were incubated with RayBiotech Mouse Inflammation Antibody Array C1 membrane (catalog AAM-INF-1) according to the manufacturer’s protocol. The Inflammation Array C1 membrane was used to evaluate CCL1, CCL11, CCL2, CCL24, CCL25, CCL, CCL5, CCL9, CSF1, CSF2, CSF3, CX3CL1, CXCL1, CXCL11, CXCL12, CXCL13, CXCL5, CXCL9, FASLG, IFN-γ, IL-10, IL-12A, IL-13, IL-17A, IL-1A, IL-1B, IL-2, IL-3, IL-4, IL-6, IL-9, LEP, TIMP1, TIMP2, TNF, TNFRSF1A, TNFRSF1B, TNFSF8, and XCL1 as well as vascular endothelial growth factor–A expression patterns according to the user manual. X-ray films were analyzed by Excel-based analysis software tools (RayBiotech) for the automatic computation of the extracted numerical data obtained from the array images.

### Human subjects.

Regarding human plasma from subjects with ARDS, this study was approved by the UAB Institutional Review Board. Patients with physical trauma who were admitted to UAB Hospital from December 2016 to December 2019 were enrolled. A total of 9 plasma samples were collected from 9 patients with severe trauma at the time of admission to hospital. Control plasma samples were obtained from 10 healthy donors (additional information in Supplemental Table 1). Patients included in the study were severely injured and met the local criteria for highest level trauma activation at UAB Hospital Level I Trauma Center. Patients were excluded if they were younger than 18 years old, or if consent was denied, not returned, or not obtainable due to the unavailability of the family. Patients fulfilled the American Thoracic Society criteria for ARDS (65). Patients were also excluded if they had a suspected inhalation injury or burns, had a bleeding diathesis, were known to take anticoagulant medications, had a known liver disease, had a known pregnancy, were incarcerated, were expected to expire within 1 hour of admission, or had a known do-not-resuscitate order prior to enrollment in the study. A blood sample (17.5 mL) was drawn after admission to the emergency department, but before fluid resuscitation after admission to the hospital. Whole blood was collected via a central line in acid citrate dextrose vacutainers. Plasma samples were separated and stored for up to 24 hours at 4°C. Next, plasma samples were frozen in liquid nitrogen and kept at −80°C for long-term storage.

### Human neutrophil isolation from blood and isolation of NETs.

Human blood samples were drawn through antecubital venipuncture from healthy volunteers into green top (heparinized) tubes 10–15 minutes before the experiment to ensure optimal neutrophil yield. The neutrophils were isolated as previously described (66). Multiple blood donors were used for the experiments throughout this study.

Five million purified neutrophils were incubated with PAO (250 nm) for 4 hours. NETs were isolated as described (67). Neutrophils were exposed to test solutions for 4 hours in a 96-well U bottom plate (Corning), after which time media were collected. Neutrophils were treated for 30 minutes with media containing PAO, and then the plate was spun down at 450*g* at 4°C. The medium was slowly pipetted out and was substituted with new medium. The substituted medium (containing NETs) was collected and concentrated up to 5 times its original volume using Microcon-10 kDa Centrifugal Filter Units with Ultracel-10 membrane (MilliporeSigma, MRCPRT010) following the manufacturer’s instructions, and the concentrate was frozen at –80°C until required for further study. A total of 180–250 ng/mL NETs (dsDNA) were used for the assays. The concentration of NETs used was equal across all groups per experiment.

### PAO preparation and exposure to neutrophils.

Stock solution (1 M) of PAO (MilliporeSigma) was prepared freshly by dissolving in 100% DMSO at 37°C. PAO was handled following all chemical safety rules and regulations approved by the UAB. Treatment of cells was done with either vehicle (control) or PAO (250 nm) diluted in the culture medium. BAPTA (10 μM), DPI (10 μM) (MilliporeSigma), 4-PBA (5 μM), ISRIB (5 μM), APB-2 (10 μM), and GSK484 (10 μM) were used in some experiments. In these experiments, cells were pretreated with different inhibitors for 30 minutes before the PAO challenge, and these treatments were then continued for specific time points mentioned in the results.

### Intracellular Ca^2+^ measurements.

The change in intracellular Ca^2+^ was measured using the Fluo-4 Direct Calcium Assay Kit (Invitrogen, Thermo Fisher Scientific, F10472) following the manufacturer’s instructions. Briefly, cells were incubated with 1x Fluo-4 Direct Calcium assay reagent solution in Fluo-4 Direct Calcium Assay Buffer media (50:50) with PAO. Following 10 minutes of incubation at 37°C, the plates were incubated at RT in the plate reader for 30 minutes.

### Primary HLMVEC isolation.

Primary HLMVECs were isolated as described (68). Briefly, human lung tissue was cut into small, approximately 1 cm^2^ pieces and washed on a 40 μm filter to remove red blood cells. Tissue was incubated with 0.2% type II collagenase (Worthington, Thermo Fisher Scientific) in RPMI containing 0.1% bovine serum albumin (BSA) for 2 hours on a roller at room temperature. While cutting lung tissue into small pieces, pleura, visible arterioles, bronchioles, and venules were avoided to prevent overgrowth with mesothelial and epithelial cells and to reduce contamination with macrovascular endothelial cells. Following collagenase digestion, the suspension was filtered on a 400 to 500 μm mesh and then a 100 μm sterile filter. The filtrate was centrifuged (250*g* for 5 minutes at room temperature). The supernatant was discarded and the resulting cell pellet resuspended in endothelial growth MV2 media (Lonza) containing 1% Penicillin–Streptomycin–Amphotericin B solution (Gibco, Thermo Fisher Scientific). An automated cell count was performed and cells were plated onto flasks precoated with 0.2% gelatin (w/v in MilliQ water, coated for 30 minutes at room temperature, excess gelatin solution was removed before cell addition) at approximately 10,000 cells/cm^2^. Cells were cultured at 37°C in the presence of 5% CO_2_. Nonadherent cells were removed after 24 hours in culture by gentle flushing with PBS over the flasks. The media was replaced every 3–4 days. At 80% confluence, cells were trypsinized to form a single-cell suspension.

Dynabeads were coated with Ulex europaeus agglutinin-1 (UEA-1) using Dynabeads FlowComp Flexi Kit as per manufacturer protocol. UEA-1 (MilliporeSigma, catalog U 4754) was used to a final concentration of 0.2 mg/mL for coating Dynabeads. UEA-1–coated beads were stored sterile at 4°C and remained active for a least 2 months. CD31 Dynabeads were purchased from MilliporeSigma. The cells were resuspended in PBS containing 0.1% BSA and 2 mm EDTA (Dynal Buffer), and 25 μL each of CD31 Dynabeads and UEA-1–coated beads were added. The cell/bead mixture was incubated on a rocker at 4°C for 20 minutes to minimize nonspecific binding. The beads were then washed in Dynal buffer and placed in a Dynal magnet. The bead-negative fluid was discarded. After repeated washing and magnetic separation, the bead-positive cells were counted and plated on 0.2% gelatin-coated tissue culture flasks at approximately 3000 cells/cm^2^ and incubated at 37°C in the presence of 5% CO_2_. Bead separation was performed over 3–5 passages of the cells until pure cobblestone cultures were obtained.

### Treatments of HLMVECs with NETs.

The concentrated (5 times) NETs (100 μL, 180–250 ng/mL) from the supernatant of neutrophils were incubated with HLMVEC monolayers (5 × 10^5^) or spheroids (5 × 10^4^ HLMVEC/spheroid) for various time points according to the experiments. Monolayer experiments for Western blotting were treated for 3, 6, and 16 hours. Electric Cell-Substrate Impedance Sensing (ECIS) studies were followed for 8 hours. The spheroids were treated for 24 hours with 180–250 ng/mL NETs.

### FITC-Dextran permeability assay.

HLMVEC monolayers were exposed to NETs (180–250 ng/mL), PAO (250 nm), or GSK484 (10 μM); to NETs neutralized with anti-NE (5 μg/mL); or to NETs neutralized with anti–Cit-H3 (5 μg/mL). One mg/mL of FITC-Dextran 4000 (MilliporeSigma) was added to the different treatment solutions. At the end of the incubation period, Transwell inserts with FITC-Dextran suspension were removed, and those containing FITC-Dextran basolateral media were measured for the fluorescence intensity at 490/520 nm excitation/emission using BioTeK Synergy Mix plate reader (Agilent Technologies).

### HLMVEC spheroid preparation and permeability assay.

HLMVEC spheroids (5 × 10^4^ HLMVECs/spheroid) were prepared and analyzed for permeability changes as described (69). Spheroids were treated with 180–250 ng/mL NETs for 24 hours.

### ECIS measurements.

ECIS was utilized to measure the electrical resistance offered by a monolayer of HLMVECs to the flow of current (Applied BioPhysics). The 8W1E array was utilized (Applied BioPhysics). Each well of the array was precoated with 0.2% gelatin followed by stabilization of the electrodes. Next, the wells were seeded with 400 μL of a monodisperse cell suspension at a concentration of 2.5 × 10^5^ cells/mL. Resistance measurements were collected at 64 kHz. Twenty hours after seeding, the array was inspected in order to ensure that all electrodes were fully covered by a monolayer of cells. Next, the cells in the array were exposed to the conditioned media with or without PAO or NETs.

### Transmission electron microscopy.

Control and PAO-treated (250 nm for 30 minutes) neutrophil samples were processed as described previously (Tecnai Spirit T12 Transmission Electron Microscope, Thermo Fisher Scientific, formerly FEI) (70).

### Immunoblot analysis.

Whole-cell lysates and concentrated supernatants harvested from neutrophils and HMVECs were processed for immunoblotting according to standard procedures using the following inhibitors or primary antibodies: PAD4 inhibitor GSK484 (Cayman Chemical), anti–Cit-H3 (Abcam, catalog ab5103), anti-PAD4 (GeneTex, catalog GTX113945), anti–ZO-1 antibody (Primary antibody, Cell Signaling Technology, catalog 13663), anti–VE-Cadherin antibody (Cell Signaling Technology, catalog 2158), anti–β-catenin (Santa Cruz Biotechnology, catalog sc-7963), anti–β-actin (Santa Cruz Biotechnology, catalog sc47778), and anti-GAPDH antibody (Cell Signaling Technology, catalog 5174). Briefly, after electrophoresis on 4%–20% gels (Bio-Rad), proteins were transferred to a PVDF membrane (MilliporeSigma). Membranes were blocked, incubated with primary antibodies as shown above, and exposed to HRP-conjugated anti-rabbit or anti-mouse antibodies (MilliporeSigma, catalog AP187P and AP130). Images were developed using a Bio-Rad system, and band intensities were quantitated using ImageJ (NIH).

### Histopathological examination and immunohistochemistry.

H&E stain was performed as described earlier (70). Briefly, lungs were fixed in 10% buffered formalin and embedded in paraffin. Tissues were cut into 5 μm sections using a microtome (HM 325, Thermo Fisher Scientific). Sections were deparaffinized in xylene and then rehydrated. At least 3 independent tissue sections from each group were stained with H&E and examined for histological alterations using a Keyance microscope.

The immunohistochemistry was performed as described (64). PAD4 antibody (GeneTex, catalog GTX113945) at dilution 1:250 and anti–citrullinated histones (MilliporeSigma, catalog 07-596) at dilution 1:200 were used for this application.

### Immunofluorescence staining of neutrophils, lung sections, and spheroids.

The collection of blood samples from a healthy human volunteer was approved by the Institutional Review Board (IRB) of the UAB. Neutrophil isolation has been described previously (67). Neutrophils were incubated in RPMI 10% containing 20 μM Hoechst 33342 for 30 minutes at 37°C, in the presence or absence of chemical inhibitors, and washed twice with RPMI 1640 (without phenol red) supplemented with 2% FBS, 50 U/mL penicillin-streptomycin, and 10 mm HEPES. A total of 5 × 10^4^ neutrophils were seeded on 0.001% poly-l-lysine–precoated (MilliporeSigma) wells of a clear bottom 96-well plate (iBidi) and challenged with 250 nm PAO (MilliporeSigma) in RPMI 2% containing 4 nm SYTOX Green (Life Technologies, Thermo Fisher Scientific). Release of NETs was imaged after 4 hours using a Nikon A1 confocal microscope.

For all other immunofluorescence staining of NETs, neutrophils were washed before fixation in 4% paraformaldehyde/PBS for 10 minutes at room temperature (RT). After 3 washes with PBS for 5 minutes each, cells were blocked in 5% donkey serum for 1 hour at RT. Neutrophils were then probed overnight at 4°C with an anti–Cit-H3 monoclonal antibody (polyclonal rabbit antibody, Abcam), NE (monoclonal mouse antibody, Abcam), anti-PAD4 (polyclonal rabbit antibody, Abcam), and anti-MPO antibody (polyclonal goat antibody, AF3667-SP, R&D Systems, Bio-Techne) at dilution 1:500 in 5% normal donkey serum. Cells were washed with PBS 3 times for 5 minutes each. Next, cells were incubated at RT for 1 hour with Alexa Fluor 488–conjugated donkey anti-rabbit and Alexa Fluor 594–conjugated donkey anti-mouse IgG or Alexa Fluor 488–conjugated donkey anti-goat (Molecular Probes, Thermo Fisher Scientific, A-21206, R37115, A-11055) at a dilution of 1:400 in 10% normal donkey serum. Following another 3 washes with PBS, the coverslips were mounted with anti-fade reagent with DAPI (Thermo Fisher Scientific).

Isolated NETs from neutrophil supernatants were stained by incubating with 4 nm TOTO 3 (Invitrogen, Thermo Fisher Scientific) for 20 minutes prior to treatment in HLMVEC spheroids. Immunofluorescence staining for spheroids was performed as described (69). Primary antibody ZO-1 (Cell Signaling Technology, catalog 13663) was used at 1:300 dilution. Images were obtained using a Nikon A1 confocal microscope.

### Study approval.

All studies were approved by the UAB IRB (IRB 110608003, IRB 300004599) for plasma samples. All animal protocols were approved by the Institutional Animal Care and Use Committee (animal protocol 21376) of the UAB.

### Statistics.

Data are expressed as the mean ± SEM. The Mann-Whitney *U* test was used for comparison between 2 distinct groups, and within-group comparisons were performed by the Wilcoxon signed-rank test. Other statistical analyses were performed by using 1-way ANOVA followed by Bonferroni’s multiple comparisons test, unless otherwise indicated. *P* < 0.05 was considered significant. All statistical analyses were performed using GraphPad Prism 8.0.

## Author contributions

RS and VBA conceived and designed the experiments, performed the experiments, analyzed the data, and wrote the manuscript. RS, MK, RKS, AMT, ZW, FJL, and PS performed the experiments. MK, RKS, RS, and ZW contributed with the animal exposure experiments. KGD, A Ahmad, A Agarwal, MA, and VBA revised the data and manuscript. JWZ and JFP provided data on ARDS patient samples. VBA conceived the project, analyzed the data, and revised the manuscript.

## Supplementary Material

Supplemental data

## Version 1 05/24/2021

Electronic publication

## Version 2 08/10/2026

After publication, the authors became aware of an error in Figure 8A.The representative higher-magnification GSK484 + PAO panel was inadvertently duplicated from the control panel during figure preparation. This has been corrected.

## References

[1] Rezoagli E (2017). Definition and epidemiology of acute respiratory distress syndrome. Ann Transl Med.

[2] Villar J (2014). The acute respiratory distress syndrome: incidence and mortality, has it changed?. Curr Opin Crit Care.

[3] Reilly JP (2019). Acute respiratory distress syndrome phenotypes. Semin Respir Crit Care Med.

[4] Belenkiy SM (2014). Acute respiratory distress syndrome in wartime military burns: application of the Berlin criteria. J Trauma Acute Care Surg.

[5] http://ameriburn.org/who-we-are/media/burn-incidence-fact-sheet/.

[6] Silva L (2016). Acute respiratory distress syndrome in burn patients: incidence and risk factor analysis. Ann Burns Fire Disasters.

[7] Shin HJ (2017). Acute respiratory distress syndrome and chemical burns after exposure to chlorine-containing bleach: a case report. J Thorac Dis.

[8] Ainsworth CR (2018). Revisiting extracorporeal membrane oxygenation for ARDS in burns: a case series and review of the literature. Burns.

[9] Calum H (2014). Burn mouse models. Methods Mol Biol.

[10] Waters LL, Stock C (1945). BAL (British anti-lewisite). Science.

[11] Sanderson H (2014). Review of environmental exposure concentrations of chemical warfare agent residues and associated the fish community risk following the construction and completion of the Nord Stream gas pipeline between Russia and Germany. J Hazard Mater.

[12] Greenberg MI (2016). Sea-dumped chemical weapons: environmental risk, occupational hazard. Clin Toxicol (Phila).

[13] Manzoor S (2020). Cutaneous lewisite exposure causes acute lung injury. Ann N Y Acad Sci.

[14] Kolaczkowska E, Kubes P (2013). Neutrophil recruitment and function in health and inflammation. Nat Rev Immunol.

[15] Abraham E (2003). Neutrophils and acute lung injury. Crit Care Med.

[16] Papayannopoulos V (2018). Neutrophil extracellular traps in immunity and disease. Nat Rev Immunol.

[17] Li P (2010). PAD4 is essential for antibacterial innate immunity mediated by neutrophil extracellular traps. J Exp Med.

[18] Lewis HD (2015). Inhibition of PAD4 activity is sufficient to disrupt mouse and human NET formation. Nat Chem Biol.

[19] Wang Y (2009). Histone hypercitrullination mediates chromatin decondensation and neutrophil extracellular trap formation. J Cell Biol.

[20] Chiu TW (2006). Plasma cell-free DNA as an indicator of severity of injury in burn patients. Clin Chem Lab Med.

[21] Kaufman T (2017). Nucleosomes and neutrophil extracellular traps in septic and burn patients. Clin Immunol.

[22] Ojima M (2020). Serial change of neutrophil extracellular traps in tracheal aspirate of patients with acute respiratory distress syndrome: report of three cases. J Intensive Care.

[23] Sakuma M (2019). Mechanism of pulmonary immunosuppression: extrapulmonary burn injury suppresses bacterial endotoxin-induced pulmonary neutrophil recruitment and neutrophil extracellular trap (NET) formation. FASEB J.

[24] Matute-Bello G (2011). An official American Thoracic Society workshop report: features and measurements of experimental acute lung injury in animals. Am J Respir Cell Mol Biol.

[25] Siemiatkowski A (2000). von Willebrand factor antigen as a prognostic marker in posttraumatic acute lung injury. Haemostasis.

[26] Munro NB (1999). The sources, fate, and toxicity of chemical warfare agent degradation products. Environ Health Perspect.

[27] Khan MA, Palaniyar N (2017). Transcriptional firing helps to drive NETosis. Sci Rep.

[28] Knuckley B (2010). Substrate specificity and kinetic studies of PADs 1, 3, and 4 identify potent and selective inhibitors of protein arginine deiminase 3. Biochemistry.

[29] Clemens RA, Lowell CA (2015). Store-operated calcium signaling in neutrophils. J Leukoc Biol.

[30] Rodrigues SF, Granger DN (2015). Blood cells and endothelial barrier function. Tissue Barriers.

[31] Moore FA (1996). Postinjury multiple organ failure: a bimodal phenomenon. J Trauma.

[32] Lee RC, Astumian RD (1996). The physicochemical basis for thermal and non-thermal ‘burn’ injuries. Burns.

[33] Altrichter J (2010). Neutrophil-derived circulating free DNA (cf-DNA/NETs), a potential prognostic marker for mortality in patients with severe burn injury. Eur J Trauma Emerg Surg.

[34] Jorch SK, Kubes P (2017). An emerging role for neutrophil extracellular traps in noninfectious disease. Nat Med.

[35] Huang H (2015). Damage-associated molecular pattern-activated neutrophil extracellular trap exacerbates sterile inflammatory liver injury. Hepatology.

[36] Yoo DG (2014). Release of cystic fibrosis airway inflammatory markers from Pseudomonas aeruginosa-stimulated human neutrophils involves NADPH oxidase-dependent extracellular DNA trap formation. J Immunol.

[37] Douda DN (2015). SK3 channel and mitochondrial ROS mediate NADPH oxidase-independent NETosis induced by calcium influx. Proc Natl Acad Sci U S A.

[38] Doussiere J (1998). Phenylarsine oxide as an inhibitor of the activation of the neutrophil NADPH oxidase--identification of the beta subunit of the flavocytochrome b component of the NADPH oxidase as a target site for phenylarsine oxide by photoaffinity labeling and photoinactivation. Eur J Biochem.

[39] Roussin A (1997). Neutrophil-associated inflammatory responses in rats are inhibited by phenylarsine oxide. Eur J Pharmacol.

[40] Wang JP (2005). Stimulation of intracellular Ca2+ elevation in neutrophils by thiol-oxidizing phenylarsine oxide. Biochem Pharmacol.

[41] Krebs J (2015). Ca(2+) homeostasis and endoplasmic reticulum (ER) stress: an integrated view of calcium signaling. Biochem Biophys Res Commun.

[42] Li C (2016). Molecular mechanism underlying pathogenesis of lewisite-induced cutaneous blistering and inflammation: chemical chaperones as potential novel antidotes. Am J Pathol.

[43] Srivastava RK (2016). Defining cutaneous molecular pathobiology of arsenicals using phenylarsine oxide as a prototype. Sci Rep.

[44] Berridge MJ (2000). The versatility and universality of calcium signalling. Nat Rev Mol Cell Biol.

[45] Maruyama T (1997). 2APB, 2-aminoethoxydiphenyl borate, a membrane-penetrable modulator of Ins(1,4,5)P3-induced Ca2+ release. J Biochem.

[46] Allam R (2013). Histones trigger sterile inflammation by activating the NLRP3 inflammasome. Eur J Immunol.

[47] Ward CM (1997). Binding of the von Willebrand factor A1 domain to histone. Thromb Res.

[48] Fuchs TA (2010). Extracellular DNA traps promote thrombosis. Proc Natl Acad Sci U S A.

[49] Grassle S (2014). von Willebrand factor directly interacts with DNA from neutrophil extracellular traps. Arterioscler Thromb Vasc Biol.

[50] Afshar M (2019). Injury characteristics and von Willebrand factor for the prediction of acute respiratory distress syndrome in patients with burn injury: development and internal validation. Ann Surg.

[51] Hirano S (2003). Difference in uptake and toxicity of trivalent and pentavalent inorganic arsenic in rat heart microvessel endothelial cells. Arch Toxicol.

[52] Doerschuk CM (1987). Marginated pool of neutrophils in rabbit lungs. J Appl Physiol (1985).

[53] Garner H, de Visser KE (2017). Neutrophils take a round-trip. Science.

[54] Peiseler M, Kubes P (2019). More friend than foe: the emerging role of neutrophils in tissue repair. J Clin Invest.

[55] Epelman S (2015). Role of innate and adaptive immune mechanisms in cardiac injury and repair. Nat Rev Immunol.

[56] Li Y (2014). Citrullinated histone H3: a novel target for the treatment of sepsis. Surgery.

[57] Biron BM (2017). Cl-amidine prevents histone 3 citrullination and neutrophil extracellular trap formation, and improves survival in a murine sepsis model. J Innate Immun.

[58] Zhao T (2016). Protective effect of Cl-amidine against CLP-induced lethal septic shock in mice. Sci Rep.

[59] Koushik S (2017). PAD4: pathophysiology, current therapeutics and future perspective in rheumatoid arthritis. Expert Opin Ther Targets.

[60] Brahmajosyula M, Miyake M (2013). Localization and expression of peptidylarginine deiminase 4 (PAD4) in mammalian oocytes and preimplantation embryos. Zygote.

[61] Ghari F (2016). Citrullination-acetylation interplay guides E2F-1 activity during the inflammatory response. Sci Adv.

[62] Sun B (2019). Reciprocal regulation of Th2 and Th17 cells by PAD2-mediated citrullination. JCI Insight.

[63] Meegan JE (2018). Citrullinated histone 3 causes endothelial barrier dysfunction. Biochem Biophys Res Commun.

[64] Surolia R (2015). Heme oxygenase-1-mediated autophagy protects against pulmonary endothelial cell death and development of emphysema in cadmium-treated mice. Am J Physiol Lung Cell Mol Physiol.

[65] Oromendia C, Siempos (2018). Reclassification of acute respiratory distress syndrome: a secondary analysis of the ARDS network trials. Ann Am Thorac Soc.

[66] Oh H Neutrophil isolation protocol. J Vis Exp.

[67] Najmeh S Simplified human Neutrophil Extracellular Traps (NETs) isolation and handling. J Vis Exp.

[68] Mackay LS (2013). Isolation and characterisation of human pulmonary microvascular endothelial cells from patients with severe emphysema. Respir Res.

[69] Cho CF (2017). Blood-brain-barrier spheroids as an in vitro screening platform for brain-penetrating agents. Nat Commun.

[70] Surolia R (2019). Vimentin intermediate filament assembly regulates fibroblast invasion in fibrogenic lung injury. JCI Insight.

