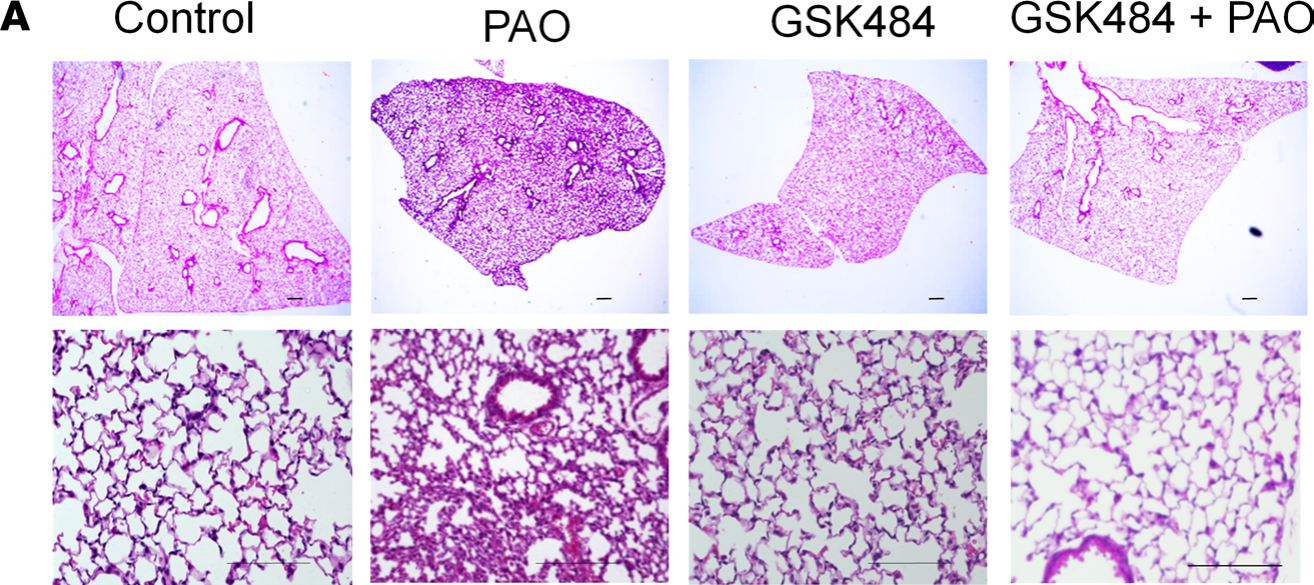# Corrigendum to NETosis in the pathogenesis of acute lung injury following cutaneous chemical burns

**DOI:** 10.1172/jci.insight.209641

**Published:** 2026-08-10

**Authors:** Ranu Surolia, Fu Jun Li, Zheng Wang, Mahendra Kashyap, Ritesh Kumar Srivastava, Amie M. Traylor, Pooja Singh, Kevin G. Dsouza, Harrison Kim, Jean-Francois Pittet, Jaroslaw W. Zmijewski, Anupam Agarwal, Mohammad Athar, Aftab Ahmad, Veena B. Antony

Original citation: *JCI Insight*. 2026;11(14):e209641. https://doi.org/10.1172/jci.insight.209641

Citation for this research: *JCI Insight*. 2021;6(10):e147564. https://doi.org/10.1172/jci.insight.147564

After publication, the authors became aware of an error in Figure 8A.The representative higher-magnification GSK484 + PAO panel was inadvertently duplicated from the control panel during figure preparation. The correct figure is shown below, and the HTML and PDF versions have been updated.

The authors regret the error.

## Figures and Tables

**Figure 8:**