# Supplementary material for: NETosis in the pathogenesis of acute lung injury following cutaneous chemical burns
Source: JCI Insight. 2021 May 24;6(10):e147564. doi: 10.1172/jci.insight.147564 (PMC8262367; doi:10.1172/jci.insight.147564)
Supplement: Supplemental data [file jciinsight-6-147564-s091.pdf]

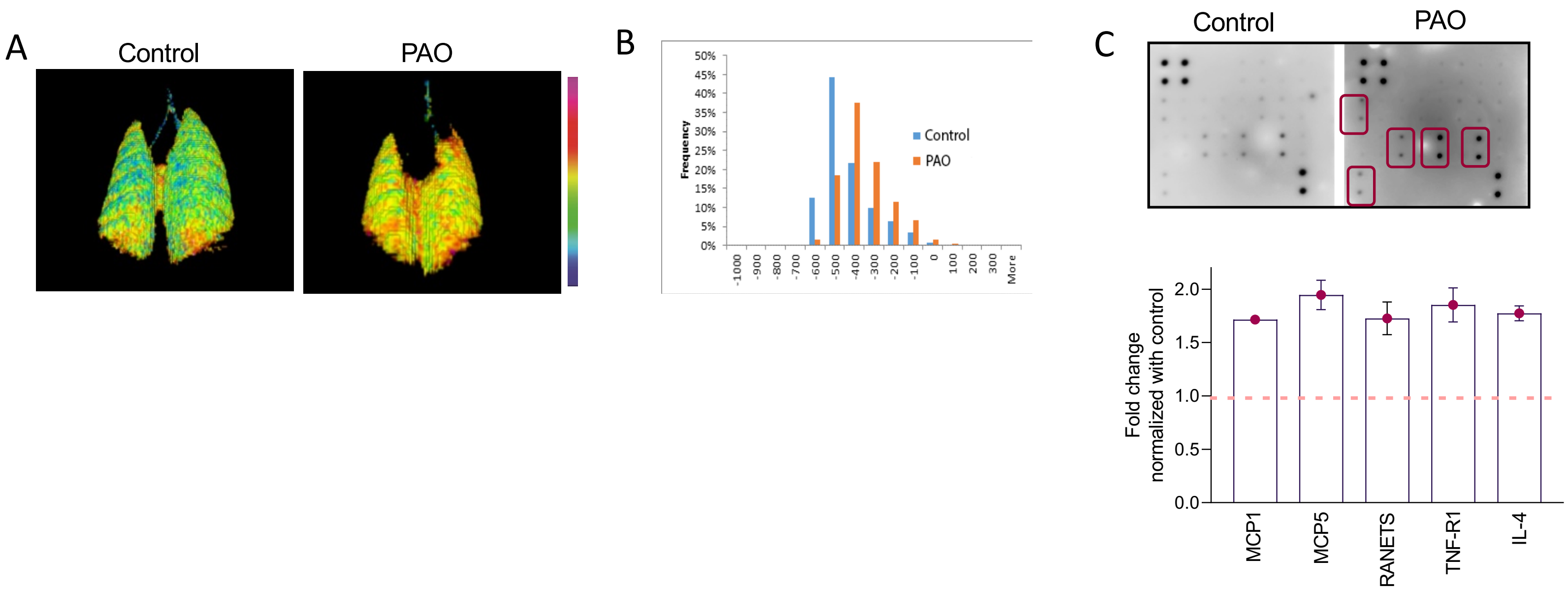

Supplementary Fig. 1: Single exposure of PAO on the skin results in acute lung injury. (**A**) 3D virtual composition of lung micro-computed tomography (CT) slice images of individual mice, from controls and PAO exposed group. The red-to-blue color scale (more density to less density) demonstrated changes in lung density. (**B**) micro-CT image signal histogram of the volume of lung tissue over the full range of tissue density for a single whole lung from a control mouse (blue bars), and PAO mouse (orange bars) (n=5-7 for A-B). (**C**) Cytokine protein array (Raybio® C series AAM-CYT-1-4) for acute inflammation in whole lung lysate of control and PAO groups. The selected cytokine levels are represented in the graph after normalization with the control group (n=3).

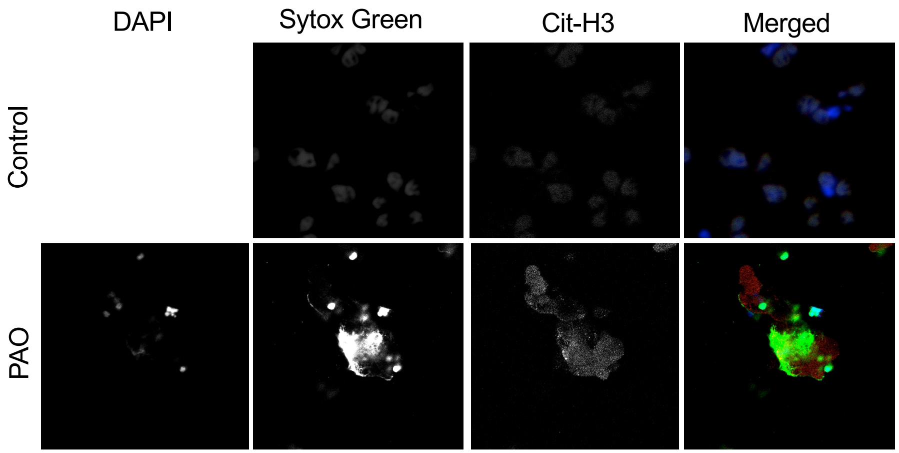

Supplementary Fig. 2: PAO treatment induces release of Cit-H3 positive NETs in human neutrophils. Neutrophils were treated with 250nM PAO for 4 hours. The un-permeablized neutrophils were stained for Sytox green (ds DNA) and Cit-H3 (tagged with Alexa-594).

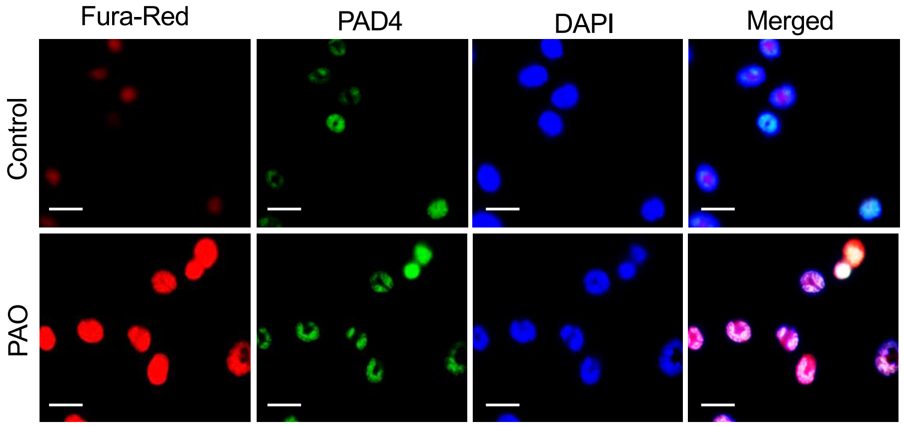

Supplementary Fig. 3: PAO treatment increases calcium and PAD4 levels in neutrophils. Human peripheral blood neutrophils were loaded with FURA-red for the detection of intracellular calcium levels (FURA-red) in controls and PAO treated group. The live neutrophils were stained for anti-PAD4-Alexa 488.

Supplementary Fig. 4

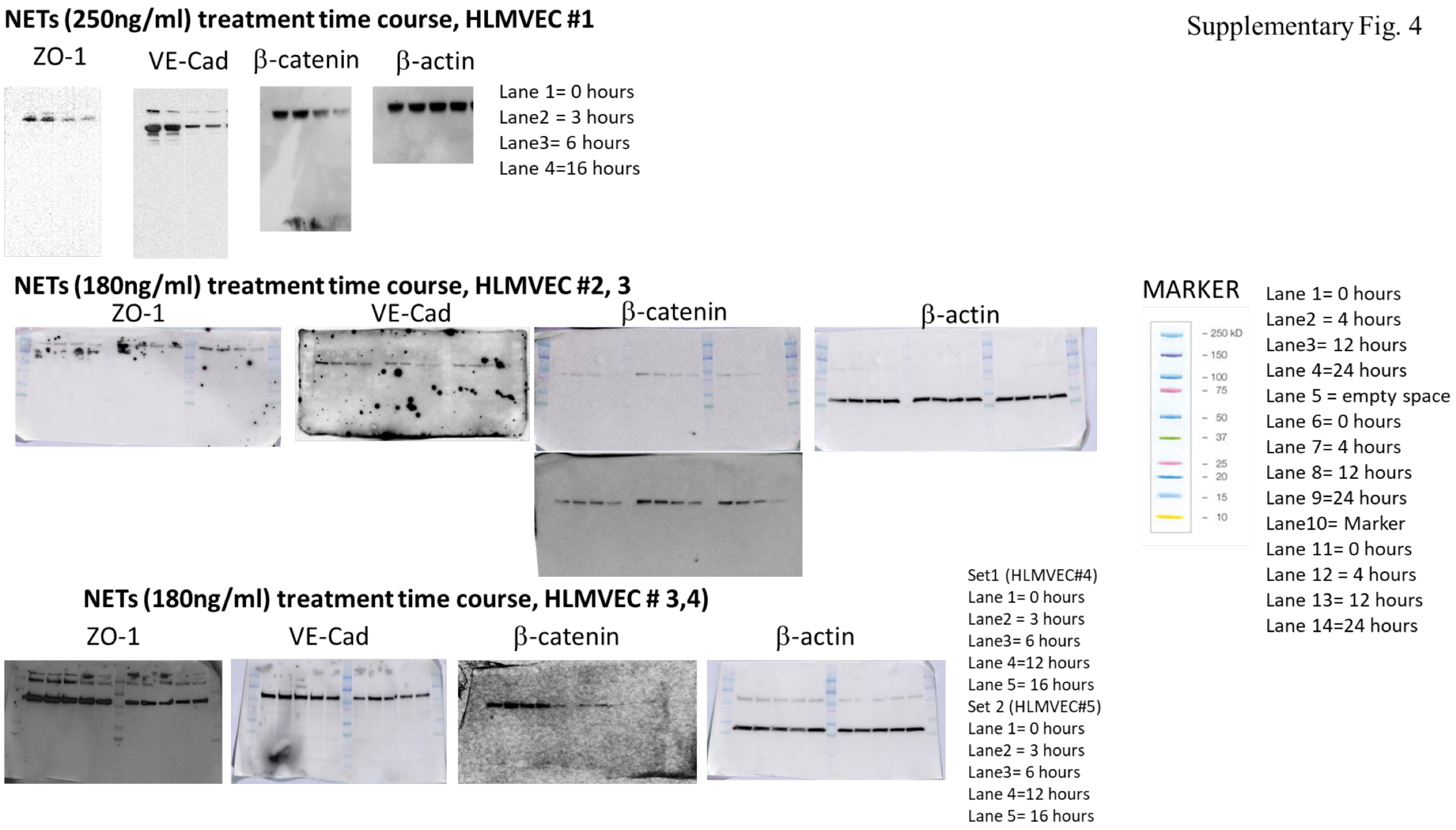

Supplementary Fig. 4: PAO treatment increases permeability of HLMVEC monolayers. HLMVECs were treated with NETs (180-250ng/ml) for indicated hours. Immunoblots of ZO-1, β-catenin, and VE-cadherin demonstrated decreased levels over the period of time upon PAO treatment. β-actin used as loading control.

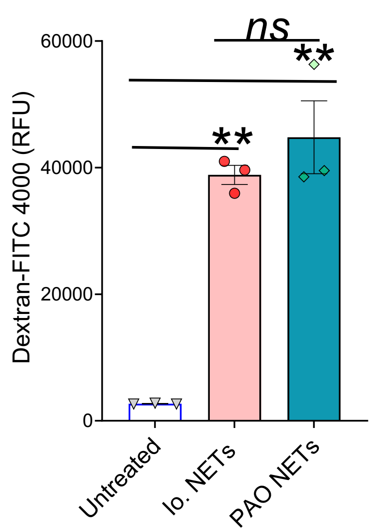

Supplementary Fig. 5: FITC Dextran permeability assay: The HLMVECs monolayers were treated with equal concentrations of NETs obtained from neutrophils treated with Ionomycin (5 µM) for 30 minutes, and PAO (250 nM) for 4 hours. Neutrophils were obtained from 3 individuals. *ns*=not significant,***P*<0.01, 1-way ANOVA followed by Tukey's multiple comparisons test

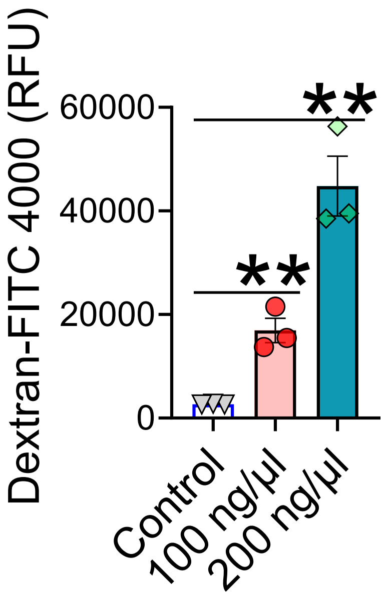

Supplementary Fig. 6: FITC Dextran permeability assay: The HLMVECs monolayers were treated with different concentrations of NETs for 24 hours. Neutrophils were obtained from 3 individuals. ***P*<0.01, 1-way ANOVA followed by Tukey's multiple comparisons test

Supplementary Table 1

ARDS patients

Case ID	Age	Sex	Race	ARDS	Pneumonia
**1**	70	Male	White	+	-
**2**	25	Male	Black	+	-
**3**	38	Male	White	+	-
**4**	59	Male	White	+	-
**5**	35	Male	Black	+	-
**6**	31	Male	White	+	-
**7**	27	Male	Hispanic	+	-
**8**	53	Female	White	+	-
**9**	48	Male	White	+	-

Healthy donors

Case ID	Age	Sex	Race
**1**	26	Female	Black
**2**	35	Female	White
**3**	23	Female	White
**4**	41	Female	White
**5**	43	Male	White
**6**	30	Male	White
**7**	27	Male	White
**8**	24	Male	Hispanic
**9**	48	Female	Black
**10**	51	Male	White

Supplementary table 1: ARDS patients demographics
